# Precise localization of metal nanoparticles in dendrimer nanosnakes or inner periphery and consequences in catalysis

**DOI:** 10.1038/ncomms13152

**Published:** 2016-10-19

**Authors:** Xiang Liu, Danijela Gregurec, Joseba Irigoyen, Angel Martinez, Sergio Moya, Roberto Ciganda, Philippe Hermange, Jaime Ruiz, Didier Astruc

**Affiliations:** 1 ISM, UMR CNRS N°5255, Univ. Bordeaux, 33405 Talence, France; 2 UMR 6226, Institut des Sciences Chimiques de Rennes, CNRS-Université de Rennes 1, Campus de Beaulieu, 35042 Rennes, France; 3 CIC biomaGUNE, Unidad Biosuperficies, Paseo Miramon No. 182, Edif ‘C', 20009 Donostia-San Sebastian, Spain; 4 Chemistry Department of San Sebastian, Basque Country University, Apdo. 1072, 20080 San Sebastian, Spain

## Abstract

Understanding the relationship between the location of nanoparticles (NPs) in an organic matrix and their catalytic performances is essential for catalyst design. Here we show that catalytic activities of Au, Ag and CuNPs stabilized by dendrimers using coordination to intradendritic triazoles, galvanic replacement or stabilization outside dendrimers strongly depends on their location. AgNPs are found at the inner click dendrimer periphery, whereas CuNPs and AuNPs are encapsulated in click dendrimer nanosnakes. AuNPs and AgNPs formed by galvanic replacement are larger than precursors and only partly encapsulated. AuNPs are all the better 4-nitrophenol reduction catalysts as they are less sterically inhibited by the dendrimer interior, whereas on the contrary CuNPs are all the better alkyne azide cycloaddition catalysts as they are better protected from aerobic oxidation inside dendrimers. This work highlights the role of the location in macromolecules on the catalytic efficiency of metal nanoparticles and rationalizes optimization in catalyst engineering.

## Abstract (web summary)

Organic supports are often employed for encapsulation of metal nanoparticles. Here the authors report a method for the precise localisation of gold, silver and copper nanoparticles within a dendrimer support and show how this localisation influences catalytic performance.

## Graphical abstract

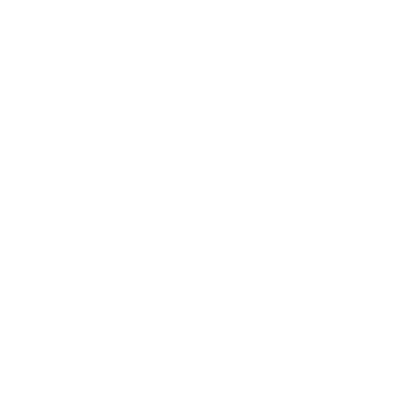

Due to their unique physical and chemical properties transition metal nanoparticles (MNPs)^1^,^2^,^3^,^4^ are being extensively used for numerous essential applications in catalysis^4^,^5^,^6^,^7^,^8^,^9^,^10^,^11^,^12^,^13^, energy storage and conversion^14^, magnetism^2^, optics^1^,^3^ and biomedicine^1^,^15^. Among many types of MNP supports, macromolecules^16^,^17^ and in particular dendrimers^9^,^18^,^19^,^20^,^21^,^22^,^23^,^24^ have attracted considerable attention because of their encapsulation properties and the possibility to guide the formation of relatively well-defined very small MNPs. It is essential, however, to research the relationship between the MNP localization in macromolecules and their catalytic properties and performances. Following the discovery of MNP stabilization by dendrimers, the seminal works by Crooks'group^9^,^18^,^19^ followed by others^20^,^21^,^22^,^23^ have disclosed catalytic, electrocatalytic and sensing properties of PAMAM dendrimer-encapsulated NPs (DENs) and the functions of dendrimers as substrate nanofilters^18^,^24^. The discovery, design and nanoengineering of click dendrimers (CD), that is, synthesized by Cu(I)-catalysed alkyne azide cycloaddition (AAC)^25^,^26^,^27^,^28^ and therefore containing intradendritic 1,2,3-triazolyl groups, has recently provoked a resurgence of interest for dendrimer-assisted MNP catalysis^29^,^30^,^31^. For instance it was shown that click-dendrimer-encapsulated PdNPs (Pd CDENs) using **1** catalyse useful cross C–C coupling reactions in only p.p.m. or sub-p.p.m. Pd amounts in aqueous media under ambient or mild conditions^31^. Although the encapsulation of MNPs inside dendrimers has been demonstrated by Crooks'groups^18^, the exact localization of MNPs in DENs still remained elusive^32^,^33^. Given the properties of MNPs^1^,^2^,^3^,^4^,^33^,^34^,^35^,^36^,^37^, the knowledge and understanding of this localization are important to optimize MNP engineering towards specific applications, and in particular catalysis. Here we report the relationship between the MNP localization and catalytic activity in 4-nitrophenol reduction (4-NP) to 4-aminophenol (4-AP) by NaBH_4_ (refs 38, 39, 40, 41, 42) and CuNP-catalysed AAC reactions^43^ of MNPs stabilized by either triazole-containing or triazole-free dendrimers. These new MNPs were synthesized following direct coordination of Cu(II), Ag(I) or Au(III) on intradendritic triazole followed by NaBH_4_ reduction and by galvanic replacement^9^,^18^ of the intradendritic CuNPs by Ag or Au upon redox reactions of the cations of the latter metals. The catalytic activities were also compared with those of group-11 MNPs that are stabilized, but not encapsulated by a structurally related ligand-free dendrimer.

Coordination of Cu(II), Ag(I) and Au(III) to intradendritic triazoles of a click dendrimer followed by reduction to metal (0) NPs is carried out here and shown by transmission electron microscopy (TEM) to form Cu, Ag and Au NPs. AgNPs are localized at the inner periphery of the dendrimer, however, whereas dendrimers containing the CuNPs and AuNPs form nanosnakes. The galvanic exchange of the CuNPs and AgNPs by AuNPs is shown to produce somewhat larger AuNPs that are not fully encapsulated inside the dendrimer. Finally CuNPs and AuNPs are also stabilized interdendritically by a related dendrimer that does not contain triazole ligands. These MNPs are characterized by UV–visible, X-ray photoelectron spectroscopy (XPS), TEM, scanning TEM (STEM), energy dispersive X-ray (EDX) and X-ray diffraction, and catalytic reactions are conducted herewith for 4-NP reduction by NaBH_4_ to 4-AP and CuACC reactions. The conclusion drawn from this localization–reactivity relationship is specific to each type of catalysis with potential application of the disclosed principles to a variety of macromolecule-stabilized nanoparticle catalysts. For 4-NP reduction by NaBH_4_ to 4-AP for which rearrangement of the AuNP surface is rate limiting, the steric effect around the AuNP surface is crucial, and catalysis by AuNPs located outside the dendrimer is shown to proceed much faster than catalysis by AuNPs stabilized at the interior of click dendrimers. Along the same line AgNPs that are also active catalysts for this reaction, although less so than AuNPs, are shown to be inactive when they are intradendritically stabilized. On the other hand for CuACC reactions, it is shown that the intradendrically protected CuNPs are much more efficient than the CuNPs stabilized interdendrically, because protection from air by the dendrimer framework is the crucial parameter that is considerably more favourable for the former CuNPs.

## Results

### Nanoparticles stabilized by a triazole-containing dendrimer

**Figure 1:**
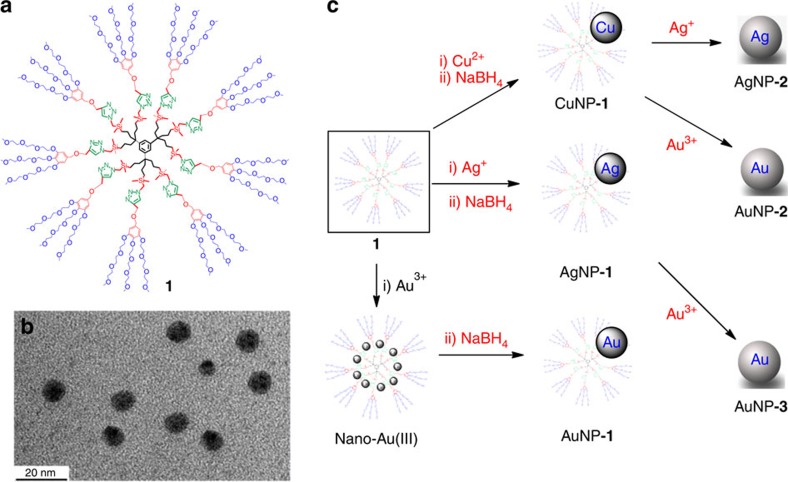
Formation of the group-11 metal NPs encapsulated and stabilized by the dendrimer 1. (**a**) Formula of dendrimer **1**; (**b**) TEM picture of the nona-Au(III) complex of **1**; (**c**) Syntheses of the metal CDENs and galvanic displacement reactions.

Dendrimer **1** (Fig. 1a) that serves as MNP template contains 27 terminal triethylene glycol groups and was previously shown by dynamic light scattering to have a 9-nm size^31^, which is confirmed here by the TEM image^33^,^34^ of its Au(III) complex synthesized by reaction of **1** with 9 equiv. HAuCl_4_·4H_2_O (Fig. 1b). The synthesis of the CDENs was conducted using water soluble salts of the metal ions and dendrimer **1** dissolved in deionized water in a standard molar ratio 1:1 of metal ion/triazole ligand followed by addition of the reductant NaBH_4_.

The CuNP galvanic displacement reactions^9^,^18^ were carried out by adding a stoichiometric amount of AgNO_3_ or HAuCl_4_·4H_2_O to an aqueous 0.1 mM solution of Cu CDEN solution to form AgNP-**2** or AuNP-**2**, or by adding a stoichiometric amount of HAuCl_4_·4 H_2_O to an aqueous 0.1 mM solution of Ag CDENs solution to form AuNP-**3** (Fig. 1c).

**Figure 2:**
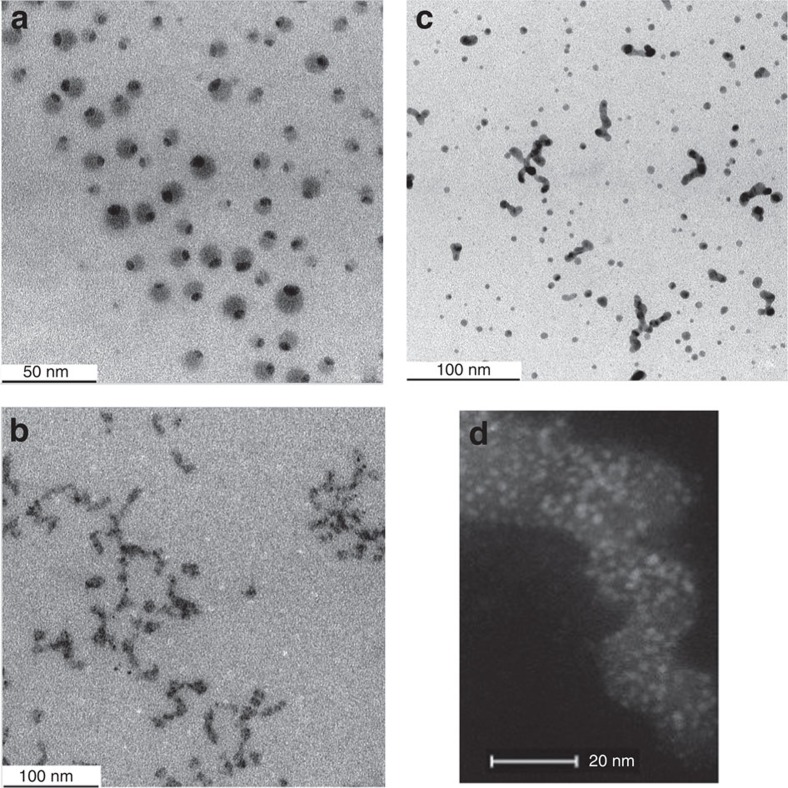
Microscopy images of the group-11 metal NPs intradendritically encapsulated by dendrimer 1. (**a**) TEM image of AgNP-**1**; (**b**) TEM image of CuNP-**1**; (**c**) TEM image of AuNP-**1**; (**d**) STEM image of CuNP-**1**.

**Figure 3:**
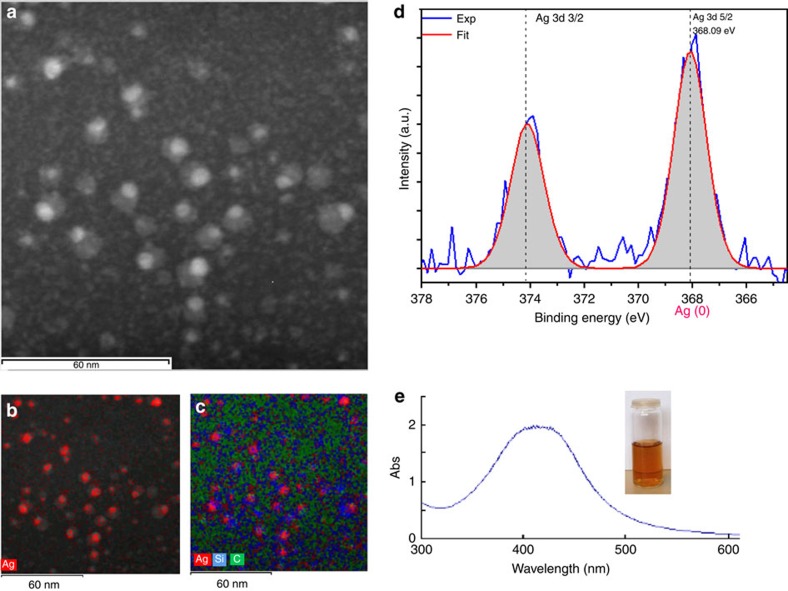
Microscopy images and spectra for AgNP-1. (**a**) STEM image of AgNP-**1**; (**b**) Ag EDX compositional map of AgNP-**1**; (**c**) combined (Ag, Si and C) EDX compositional map; (**d**) XPS of AgNP-**1**; (**e**) UV–visible spectrum (SPB at 425 nm) and photograph of AgNP-**1**.

The TEM picture shows encapsulation of the Cu, Ag and Au nanoparticles, but interestingly the case of the AgNPs is different from those of the CuNPs and AuNPs, that is, these AgNPs-**1** are localized at the inner periphery of the dendrimer **1** (Fig. 2a), whereas the dendrimers **1** that encapsulate CuNPs-**1** and AuNPs-**1** form nanosnakes (Fig. 2b,c). The localization of the AgNPs-**1** at the inner periphery of **1** is also visible in the STEM image of AgNP-**1** (Fig. 3a). The dendrimers that encapsulate the AgNP-**1** also have a size of 9 nm (TEM, grey spheres in Fig. 2a and Supplementary Fig. 1) matching the size of **1** (dynamic light scattering)^31^ and its Au(III) complex (Fig. 1b; Supplementary Fig. 2) and Ag(I) (Supplementary Fig. 3) complexes. The CDEN AgNP-**1** located at the inner periphery of **1** have a core size of 3.5 nm and a surface plasmon band (SPB) at 425 nm in the UV–visible spectrum (Fig. 3e). This size corresponds to 1,340 Ag atoms, which means that many dendrimers **1** do not contain AgNPs. This is verified in the EDX analysis compositional map of Ag by the presence of large black areas (Fig. 3b) and in the combined (Ag, C and Si) compositional map by the large green and blue dendrimer zones representing carbon resp. silicon atoms without the red spots representing the AgNPs (Fig. 3c). Such a situation is characteristic of Ostwald ripening^44^, that is, of migration of Ag atoms among dendrimers and contrasts with the previous formation of click Pd CDEPs without significant Pd atom escape^29^. It may also be specific of click dendrimers terminated by triethylene glycol groups for these metals, whereas the well-known PAMAM DENs were shown by Crooks' group to contain essentially all group-10 and group-11 metal atoms generated by reduction of their amine-coordinated cations inside the PAMAM dendrimers^18^. To complete the characterization AgNP-**1**, XPS (Fig. 3d) was recorded, showing peaks at 368.1 and 374.5 eV that correspond to 3*d*_5/2_ and 3*d*_3/2_ core levels of metallic silver, respectively. The binding energy of 368.1 eV for Ag 3*d*_5/2_ assigned to AgNPs shows that the surface atoms of AgNP-**1** are exclusively zero valent.

CuNP-**1** and AuNP-**1** also follow this same trend involving Oswald ripening as the size of their CDENs formed here by direct intradendritic triazole coordination followed by NaBH_4_ reduction have sizes of 2.0 and 4.5 nm, respectively (Supplementary Figs 4 and 5). Their TEM images are reproducibly in sharp contrast with that of AgNP-**1**, as these dendrimers appear to form nanosnake networks that encapsulate the MNPs (Fig. 2b,c). The triethylene glycol termini may play the role of a supramolecular glue binding the dendrimers to one another although monodimensionality is preferred in contrast to the dendrimers encapsulating AgNP-**1** that do not agglomerate. The STEM image of CuNP-**1** shown in Fig. 2d confirms that the CuNPs are encapsulated by nanosnake networks formed by the dendrimer molecules.

Galvanic replacement of CuNP by more noble metals^18^, that is, metals that have more positive oxidation potentials, was achieved by reactions of the Cu CDENs with the salts of these noble metals and led to 10.9 nm sized dendrimer-stabilized AgNP-**2** (Supplementary Fig. 6) and 5.1 nm sized AuNP-**2** (Supplementary Fig. 7), respectively, whereas replacement of AgNP-**1** by Au carried out by reaction with HAuCl_4_·4H_2_O led to 4.5 nm-sized dendrimer-stabilized AuNP-**3** (Supplementary Fig. 8). The fact that AgNP-**2** is much larger than CuNP-**1** shows that redox exchange proceeds along with considerable NP rearrangement and that the resulting AgNP-**2** are no longer encapsulated in the dendrimer **1**, but stabilized by several dendrimer molecules. The galvanic exchange producing AuNP-**2** and AuNP-**3** proceeds with some size increase so that the resulting AuNPs cannot be fully encapsulated inside the dendrimer. This situation contrasts with that encountered with PAMAM DENs that undergo *in situ* displacement reactions^9^,^18^.

### Nanoparticles stabilized by a triazole-free dendrimer

**Figure 4:**
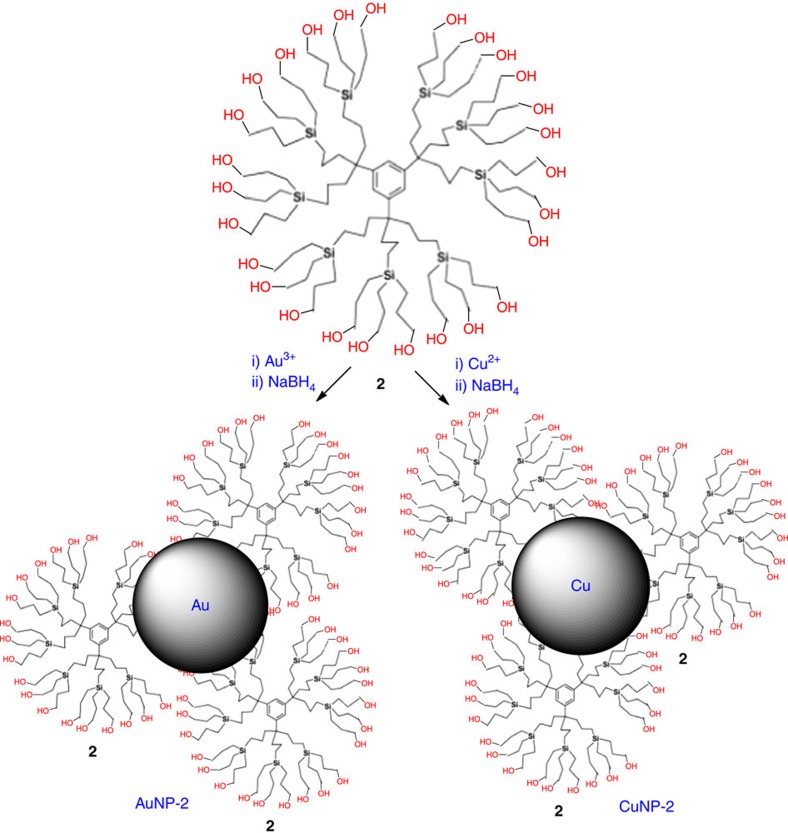
Synthesis of AuNP-2 and CuNP-2. The lack of intradendritic ligands in dendrimer **2** induces the formation of large TMNPs AuNP-**2** and CuNP-**2** stabilized by clusters of dendrimers.

To compare the behaviour of DENS with that of NPs stabilized outside dendrimers, the new water-soluble dendrimer **2** (Fig. 4) was synthesized from the same nona-allyl core that led to **1** (for more information, see Supplementary Methods). Thus dendrimer **2** resembles **1** with 27 termini but does not contain ligands, unlike **1**. Stabilization of CuNP-**2** (4.3 nm) and AuNP-**4** (6.6 nm) by dendrimer **2** upon NaBH_4_ reduction of the group-11 metal cations then occurs by interactions with several dendrimer molecules (Fig. 4). The core sizes and SPBs of all the NPs stabilized by dendrimer **1** or dendrimer **2** are gathered in Table 1.

**Figure 5:**
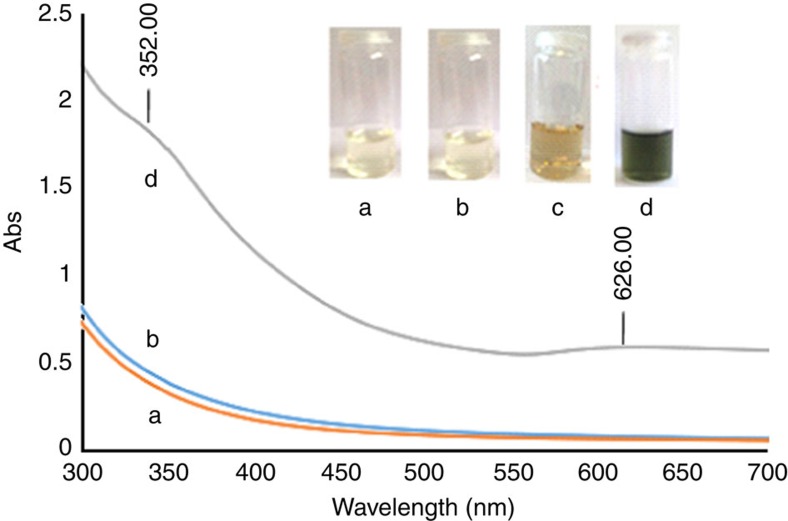
Compared photographs and UV–visible spectra of CuNP-1 and CuNP-2 upon standing in air. (a) Photograph and UV–visible spectrum of CuNP-**1** after 1 min; (b) photograph and UV–visible spectrum of CuNP-**1** after 2 months; (c) photograph of CuNP-**2** after 1 min; (d) photograph and UV–visible spectrum of CuNP-**2** after 5 min.

The goal of these syntheses was to compare the catalytic performances of CuNP-**2** (Supplementary Fig. 9) and AuNP-**4** (Supplementary Fig. 10) located outside dendrimer **2**, with the NPs that are encapsulated in the dendrimer interior of **1** or partly penetrating into **1**. CuNP-**2** is considerably more easily oxidized than the CDEN CuNP-**1** to Cu_2_O outside **2** as indicated by fast colour change from light yellow (Fig. 5c) to dark green (Fig. 5d) and the new absorption at 352 nm characteristic of Cu_2_O (ref. 45) appearing after 5 min (Fig. 5d). This oxidability of CuNP-**2** indeed greatly contrasts with that of CuNP-**1** that is fully stable as Cu(0) for at least two months as shown by UV–visible (Fig. 5a,b).

**Figure 6:**
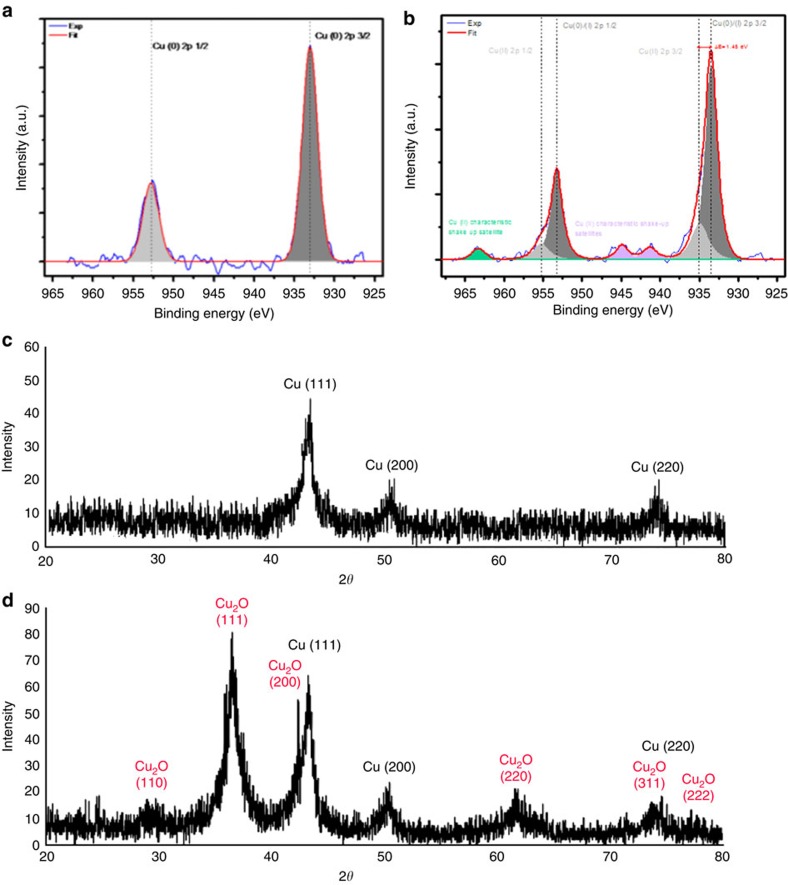
XPS and X-ray diffraction spectra of CuNP-1 and CuNP-2. (**a**) XPS spectrum of CuNP-**1** showing its long-term aerobic stability as Cu (0); (**b**) XPS spectrum of CuNP-**2** showing its oxidation as Cu (I) and Cu (II); (**c**) X-ray diffraction spectrum of CuNP-1 showing that only Cu (0) is present; (**d**) X-ray diffraction spectrum of CuNP-**2** showing the presence of both Cu (0) and Cu_2_O.

The presence of only Cu(0) in CuNP-1 is confirmed by the XPS as X-ray diffraction spectra. In XPS, CuNP-**1** (Fig. 6a) does not show shake up features characteristic of oxidized Cu at 943 and 963.5 eV. So the XPS spectrum clearly confirms Cu(0) chemical state alone. If Cu (I) would be present, a weak satellite features would be recorded at these energies, which is not the case here^46^. The X-ray diffraction of CuNP-**1** (Fig. 6c) shows its long-term aerobic stability as Cu(0). The finding that CuNP-**1** are at the oxidation state Cu(0) is logical, because their surface is only bonded to neutral triazole ligands, and it just means that the dendrimer framework is protecting inside the dendrimer interior against intradendritic aerobic CuNP oxidation. On the other hand, CuNP-**2** appears to be in the form of a mixture of Cu(0), Cu_2_O and CuO. Indeed, in the XPS spectrum of CuNP-**2** (Fig. 6b) is resolved to at least two states. The main peak at 933.4 and 952.0 eV correspond to 2*p*_3/2_ and 2*p*_1/2_ core levels of Cu(0). The pronounced shake up features at binding energies of 941.29 and 944.93 eV shows oxidation to Cu(I) or (II)^47^. Moreover Cu(II) is confirmed by detailed examination of line shape and shake up satellite feature found at 963.25 eV being characteristic of only Cu (II) thus confirming oxidation of Cu NP-2 all the way to CuO (for detailed discussion, see Supplementary Discussion).^46^ This spectrum also shows peaks at 933.4 and 952.0 eV that correspond to 2*p*_3/2_ and 2*p*_1/2_ core levels of Cu(0). The X-ray diffraction spectrum of CuNP-**2** is shown in Fig. 6d. The peaks (1 1 0) (29.5 eV), (1 1 1) (36.4 eV), (2 0 0) (42.3 eV), (2 2 0) (61.6 eV) and (3 1 1) (73.8 eV) belong to Cu_2_O, and the other peaks at (111) (43.5 eV), (200) (50.4 eV) and (220) (74.0 eV) planes belong to metallic Cu (0). CuNP-**2** are not protected inside the dendrimer and are thus considerably more exposed to aerobic oxidation as witnessed by the dramatic fast colour change within 5 min and the above spectroscopic data. This means that the inside of CuNP-**2** remain in the Cu(0) state, whereas the exposed surface Cu atoms are oxidized to Cu_2_O and CuO.

### Catalysis

**Figure 7:**
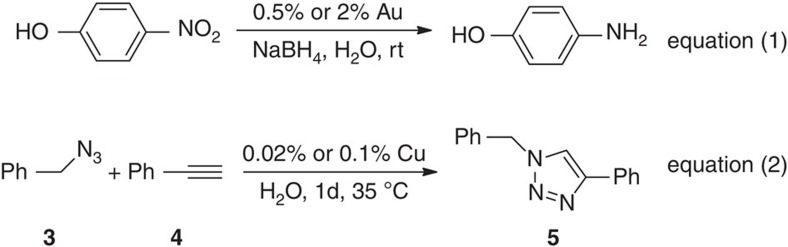
Catalytic reactions conducted with the AuPs and CuNPs as catalysts. In equation (1), the 4-NP reduction to 4-AP by NaBH_4_ is catalysed by AuNPs, and in equation (2) the copper-catalysed azide alkyne cycloaddition (CuAAC, click reaction) is catalysed by CuNPs.

To compare the catalytic properties of these CDENs with those of NPs that are not encapsulated in the dendrimer **1**, but stabilized by several molecules of dendrimer **1**, two catalytic reactions have been conducted in water: (i) the AuNP-catalysed 4-NP reduction to 4-AMP (Fig. 7, equation (1)), and (ii) the CuNP-catalysed AAC ‘click' reaction selectively giving a 1,4-diphenyl-1,2,3-triazole (Fig. 7, equation (2)).

First, the AuNP-catalysed 4-NP reduction is representative^38^,^39^,^40^,^41^,^42^, being highly sensitive to the catalyst surface environment with the Langmuir-Hinshelwood mechanism highlighted by Ballauff's group^38^,^39^. In this mechanism, both reactant molecules, 4-nitrophenol and borohydride, are adsorbed on adjacent sites of the NP surface before the reaction, and this reaction takes place via an activated complex on the NP surface involving NP surface reconstruction. Its kinetics is conveniently monitored by UV–visible spectroscopic analysis with absorptions at *λ*=400 and 300 nm corresponding to 4-NP and 4-AP, respectively, and characterized by the disappearance of the yellow colour of 4-NP upon reduction. This reaction has been carried out with 100 equiv. of NaBH_4_ in water in the presence of 0.5 mol% resp. 2.0 mol% AuNP-**1** (as shown in Supplementary Table 1). This catalysis by AuNP-**1** was found to be slow and to require an induction time of 480 s resp. 180 s due to the reorganization at the catalyst surface. This induction time is taken into account by the encapsulation of the AuNP-**1** (CDENs) inside nanosnakes (Fig. 2c) that provides partial steric inhibition of the substrate access to the AuNP surface^38^,^39^,^40^. On the other hand AuNP-**4** located outside dendrimer **2** without steric inhibition of the dendrimer encapsulation clearly benefits from easier access to the AuNP surface. Indeed no induction time is found, and the 4-NP rate constant is large. The cases of dendrimer-**1**-stabilized AuNP-**2** and AuNP-**3** are intermediate between those of AuNP-**1** and AuNP-**4**. They partly penetrate dendrimer **1** in contact with the intradendritic triazoles, but they are too large for full intradendritic encapsulation into a single dendrimer. They exhibit part of their surface outside dendrimer **1** allowing more efficient catalysis than inside dendrimer **1** but less efficient catalysis than AuNP-**4** that is not attracted by triazole ligands in the dendrimer interior. Along this line and accordingly, CuNP-**1** and AgNP-**1** that are CDENs do not catalyse 4-NP reduction under these conditions even in the presence of high catalyst amounts (the yellow colour of the solution does not disappear in two months), obviously also due to steric inhibition resulting from the encapsulation, whereas other CuNPs and AgNPs are known 4-NP reduction catalysts under ambient conditions^41^,^47^.

The second test reaction allowing a key comparison between the dendrimer-stabilized MNP catalysts—the CuAAC reaction between **3** and **4** selectively yielding **5** (Fig. 7, equation (2))^28^—was conducted using either the CDEN CuNP-**1** or the dendrimer-stabilized CuNP-**2** as catalyst.

Optimized reaction between **3** (0.55 mmol) and **4** (0.5 mmol) catalysed by CuNP-**1** (200 resp. 1,000 p.p.m. Cu) and 2 ml H_2_O for 24 h at 35 °C under N_2_ yielded 45% resp. 100% of **5**, but with CuNP-**2** the catalytic activity is poor, the yield being only 9% resp. 15% (equation (2) and Supplementary Fig. 11). This difference is best taken into account by the fact that the click reaction is strongly activated by intradendritic triazole ligands, whereas for CuNP-**2** outside dendrimer **2** and without triazole ligands no activation occurs. Interestingly note that CuNP-**1** has a pure Cu(0) state (*vide supra*), whereas for CuNP-**2**, the oxidation state I that was observed (*vide supra*) is usually favourable for CuAAC catalysis. There are a number of precedents for Cu metal or CuNP AAC catalysis^43^, and the present data confirm the efficiency of non-oxidized, but triazole-liganded Cu(0) nanoparticles. In this type of catalysis, the intradendritic protection and ligand activation are essential to inhibit the formation of oxidized CuNPs outside dendrimers and activate CuAAC catalysts.

## Discussion

The new CuNP-**1** and AuNP-**1** that are encapsulated by ‘click' dendrimer **1** terminated by triethylene glycol groups have been shown to form nanosnakes for Cu and Au, the triethylene glycol termini providing supramolecular interdendritic 1D interactions that act as a glue. On the other hand, remarkably, AgNP-**1** are localized at the inner periphery of click dendrimers. Their galvanic replacement reactions yield AuNP-**2**, AuNP-**3** and AgNP-**2** that are larger and no longer encapsulated in dendrimers but only partly penetrating into the dendrimers. With a similar triazole-free dendrimer **2**, CuNP-**2** and AuNP-**4** are only stabilized outside the dendrimer.

A correlation is then established between the catalytic activities of these MNPs and their localization, that is, inside versus partly outside the click dendrimer **1** or outside the non-click dendrimer **2**. The encapsulation of these Cu, Ag and Au NPs inside the click dendrimer **1** sterically inhibits the access to the AuNP-**1** surface slowing down the catalysis of 4-NP reduction and provoking an induction time for the reorganization of the NP surface environment. Under identical conditions, AuNP-**2** and AuNP-**3** resulting from galvanic replacement and located partly outside the click dendrimers and are less subjected to steric inhibition nor catalytic induction time and are better 4-NP reduction catalysts. Finally, AuNP-**4** stabilized outside the non-click dendrimer are the most efficient catalysts as a result of minimal steric inhibition of the catalyst surface.

On the contrary, catalysis of the CuAAC ‘click' reaction by intradendritically encapsulated and triazole-activated CuNP-**1** is efficient, providing quantitative yield of the 1,4-disubstituted triazole with 0.1 mol% Cu, because they are protected from aerobic oxidation, whereas CuNP-**2** located outside dendrimers are very rapidly oxidized at their surface and are a very poor click catalyst. In this case, the catalytic reactivity is directed by the system ability to prevent the first-raw transition metal NPs from oxidation. On the other hand, air stable AuNPs are not sensitive to this parameter, and their activity is only linked to the intradendritic steric surface inhibition. This type of correlation of NP localization vs. catalytic activity in nanomaterials, that is specific to the type of reaction and of MNPs, is essential for MNP catalyst engineering towards a greener chemistry^48^.

## Methods

### Preparation of NPs encapsulated in the dendrimer **1**

3.6 × 10^−4^ mmol of dendrimer **1** (2.59 mg) is dissolved in 1.1 ml of water in a Schlenk flask, and a colourless solution of CuSO_4_·5H_2_O, AgNO_3_ or HAuCl_4_·4H_2_O (3.2 × 10^−3^ mmol in 1.1 ml water) is added to the solution of the dendrimer. Thirty millilitre of water is added, and the solution is stirred for 1 h. The concentration of Cu(II), Ag(I) or Au(III) is 0.1 mM. A 1-ml aqueous solution containing 3.2 × 10^−2^ mmol of NaBH_4_ is added dropwise, provoking the formation of a yellow (CuNP-**1**), orange (AgNP-**1**) or purple (AuNP-**1**) colour (Fig. 2c) corresponding to the reduction of the cation to the zero-valent metal and MNP formation. A plasmon band is observed in the UV–visible spectra only for AgNP-**1** (425 nm) and AuNP-**1** (512 nm).

### Preparation of dendrimer–MNPs by galvanic displacement

A concentration of 3.6 × 10^−4^ mmol of dendrimer **1** (2.59 mg) is dissolved in 1.1 ml of water in a Schlenk flask, and a colourless solution of CuSO_4_·5H_2_O (3.2 × 10^−3^ mmol in 1.1 ml water) is added to the solution of the dendrimer. Thirty millilitre of water is added, and the solution is stirred for 1 h. The concentration of Cu(II) is 0.1 mM. A 1 ml aqueous solution containing 6.4 × 10^−3^ mmol of NaBH_4_ is added dropwise, provoking the formation of a yellow colour (Fig. 2b) corresponding to the reduction of Cu(II) to Cu(0) and formation of CuNP-**1**. The dendrimer **1**-encapsulated CuNP-**1** solution is purged with N_2_ continuously from 15 min before addition of NaBH_4_ until 2 h after reduction. A borohydride destruction procedure follows by addition of aqueous HClO_4_, the pH of the resulting 0.1 mM CuNP solution being adjusted to 3. Then a colourless solution of AgNO_3_ (3.2 × 10^−3^ mmol in 1.1 ml water) is added dropwise to the solution of the dendrimer-**1**, provoking the formation of an orange colourcorresponding to the reduction of Ag(I) to Ag(0) by CuNP-**1**. The formation of AgNP-**2** is characterized by a plasmon band at 425 nm in the UV–visible spectrum. AuNP-**2** and AuNP-**3** are similarly synthesized by galvanic displacement from CuNP-**1** resp. AgNP-**1**.

### Preparation of AuNP-4 and CuNP-2 stabilized by dendrimer 2

A concentration of 3.6 × 10^−4^ mmol of dendrimer **2** (0.8 mg, 3.6 × 10^−4^ mmol) is dissolved in 1.1 ml of water in a Schlenk flask, and a colourless solution of CuSO_4_·5H_2_O or HAuCl_4_·4H_2_O (9.72 × 10^−3^ mmol in 1.1 ml water) is added to the solution of the dendrimer. Thirty millilitre of water is added, and the solution is stirred for 1 h. The concentration of Cu(II) or Au(III) is 0.1 mM. A 1-ml aqueous solution containing 9.72 × 10^−2^ mmol of NaBH_4_ is added dropwise, provoking the formation of a green (CuNP-**2**) or purple (AuNP-**4**) colour (Supplementary Fig. 10) corresponding to the reduction of the cation to the zero-valent metal and MNP formation. A plasmon band is observed in the UV–visible spectra only for CuNP-**2** (352* nm, 628 nm) (Supplementary Fig. 9) and AuNP-**4** (520 nm) (Supplementary Fig. 10).

### Catalysis of 4-nitrophenol reduction

An aqueous solution (2.5 ml) containing 4-nitrophenol (0.09 mmol) and NaBH_4_ (7.2 mmol) is prepared in a standard quartz cuvette (3 ml, path length: 1 cm). The AuNP catalyst (0.5 mol%, 0.45 × 10^−3^ mmol) is injected into this solution, and the reaction progress is detected by UV–visible spectroscopic analysis every min at 22 °C (Supplementary Figs 12–19).

### CuAAC reaction leading to the triazole derivative **5**

Benzyl azide (**3**) 0.5 mmol, ethynylbenzene (**4**) 0.55 mmol, CuNP-**2** (0.1% mmol) and 2 ml H_2_O are taken in a round bottom flask equipped with a magnetic stirrer. The resulting mixture is stirred for 24 h at 35 °C under N_2_. After cooling to room temperature, water (2 ml) is added to the reaction mixture, and the latter is extracted with ethyl acetate (3 × 10 ml). The combined organic phases are washed with brine (2 × 5 ml), dried over anhydrous MgSO_4_ and concentrated *in vacuo*. The residue is subjected to flash column chromatography with hexanes/EtOAc (v/v=5/1) as eluent to obtain the desired 1-benzyl-4-phenyl-1H-1,2,3-triazole (**5**) as a white solid (100% yield).

### Data availability

All data is available from the authors upon reasonable request.

## Additional information

**How to cite this article:** Liu, X. *et al*. Precise localization of metal nanoparticles in dendrimer nanosnakes or inner periphery and consequences in catalysis. *Nat. Commun.* **7,** 13152 doi: 10.1038/ncomms13152 (2016).

## Supplementary Material

Supplementary Information Supplementary Figures 1-25, Supplementary Table 1, Supplementary Discussion, Supplementary Methods and Supplementary References.

## Figures and Tables

**Table 1:** SPBs and core sizes of all the NPs.

**MNPs**	**SPB (nm)**	**Diameter (nm)**
CuNP-**1**	—	2.0±0.1
CuNP-**2**	352*, 626	4.3±0.1
AgNP-**1**	425	3.5±0.1
AgNP-**2**	425	10.9±0.2
AuNP-**1**	512	4.5±0.1
AuNP-**2**	540	5.1±0.1
AuNP-**3**	527	4.5±0.1
AuNP-**4**	520	6.6±0.2

^*^The band at 352 nm is due to Cu_2_O (ref. 45).
