# Supplementary material for: Precise localization of metal nanoparticles in dendrimer nanosnakes or inner periphery and consequences in catalysis
Source: Nat Commun. 2016 Oct 19;7:13152. doi: 10.1038/ncomms13152 (PMC5075800; doi:10.1038/ncomms13152)
Supplement: Supplementary Information — Supplementary Figures 1-25, Supplementary Table 1, Supplementary Discussion, Supplementary Methods and Supplementary References. [file ncomms13152-s1.pdf]

Supplementary Figures

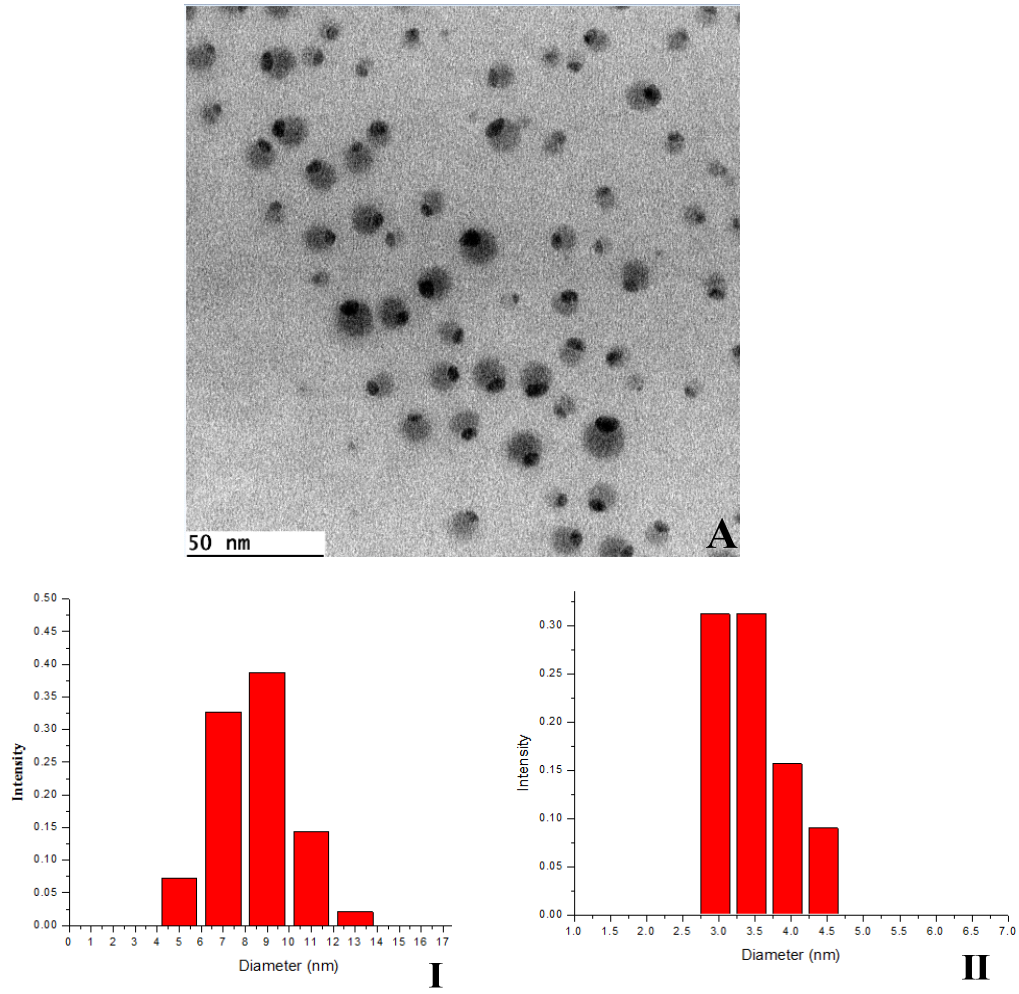

Supplementary Figure 1. A: TEM image of AgNP-**1.**

I: Diameter histogram distribution of grey spheres of AgNP-**1** (9 ± 0.2 nm).

II: Diameter histogram distribution of AgNP-**1** (3.5 ± 0.1 nm)**.**

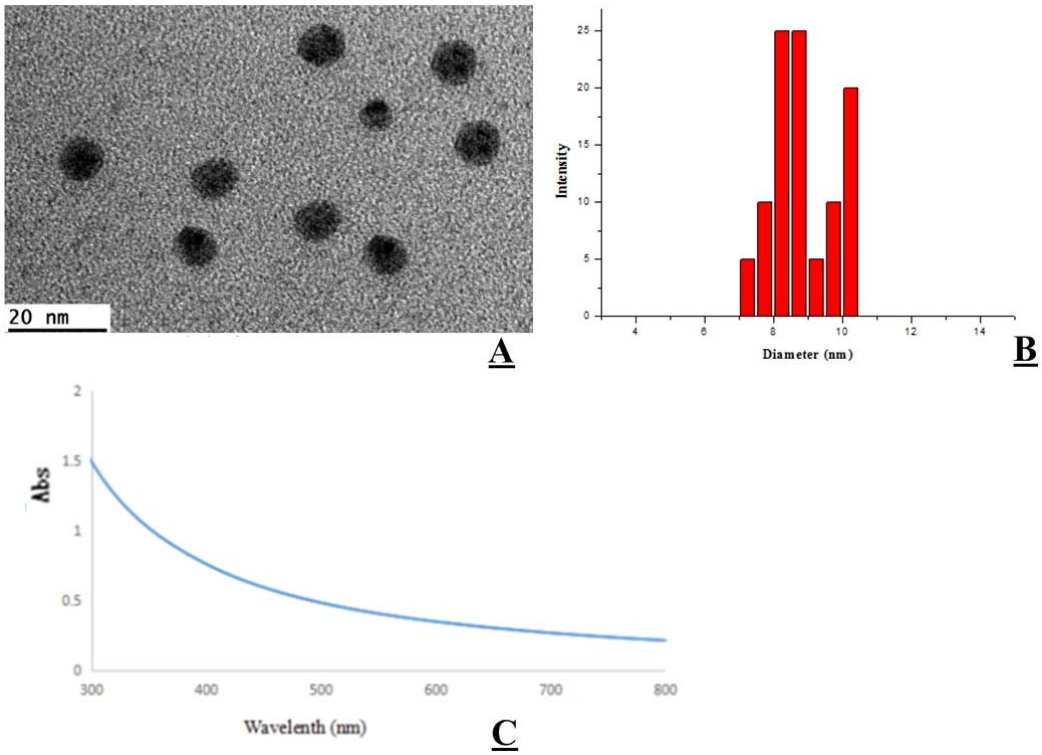

Supplementary Figure 2. A: TEM image of Au^{III}@dendrimer **1**,

B: Diameter histogram distribution of Au^{3+}@dendrimer **1** (9 ± 0.2 nm).

C : UV-vis. spectrum of Au^{III}@dendrimer **1**.

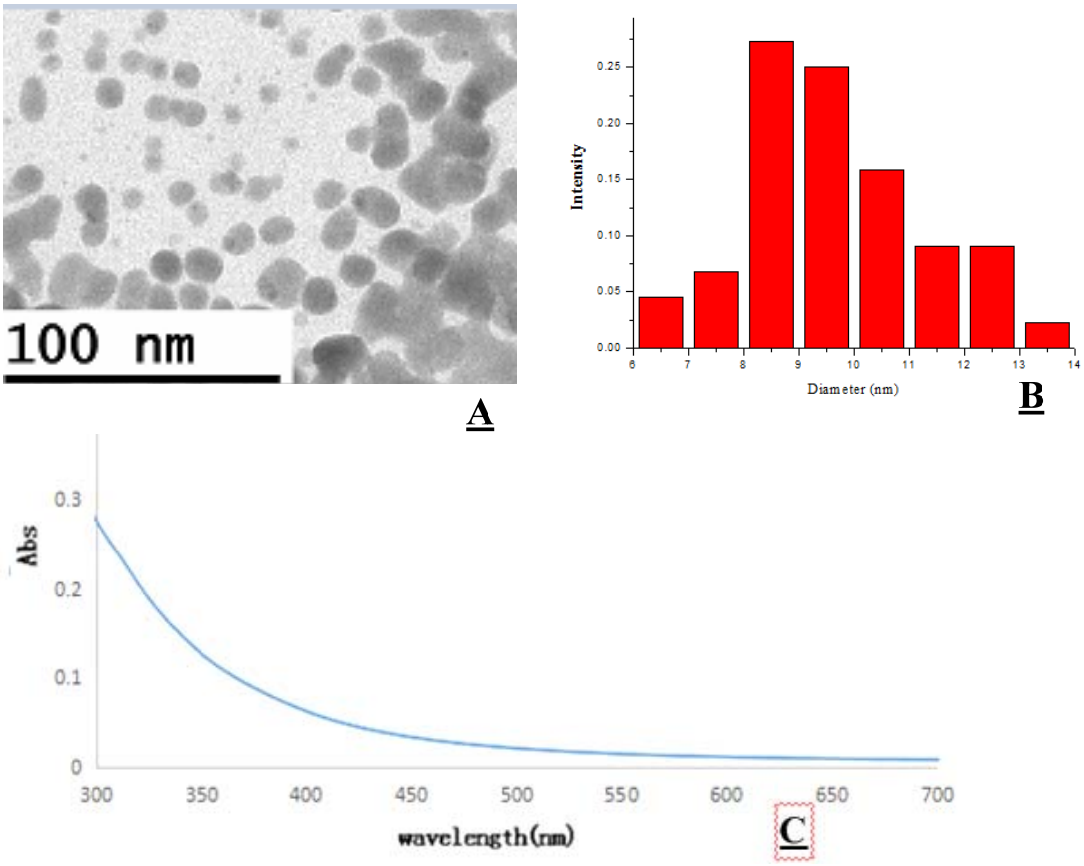

Supplementary Figure 3. A: TEM image of Ag^{+}@dendrimer **1**,

B: Diameter histogram distribution of Ag^{+}@dendrimer **1** (9 ± 0.1 nm).

C : UV-vis. spectrum of Ag^{I}@dendrimer **1**.

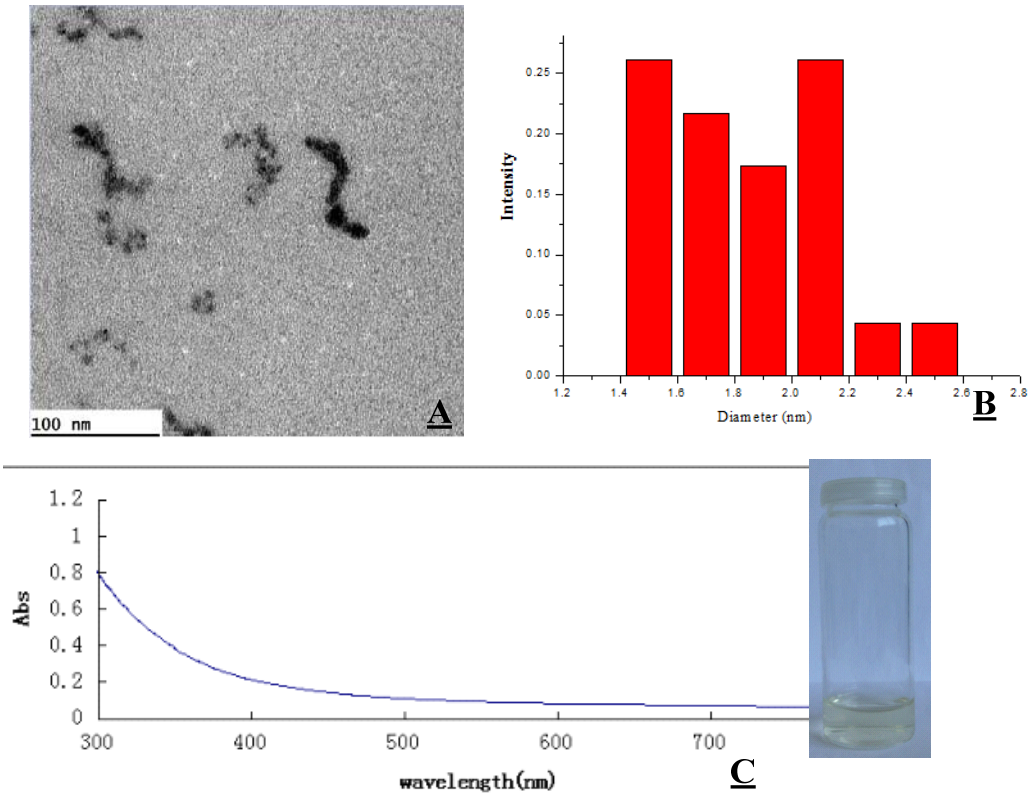

Supplementary Figure 4 A: TEM image of CuNP-**1**,

B: Diameter histogram distribution of CuNP-**1** (2.0 ± 0.1 nm).

C : UV-vis. spectrum of CuNP-**1**.

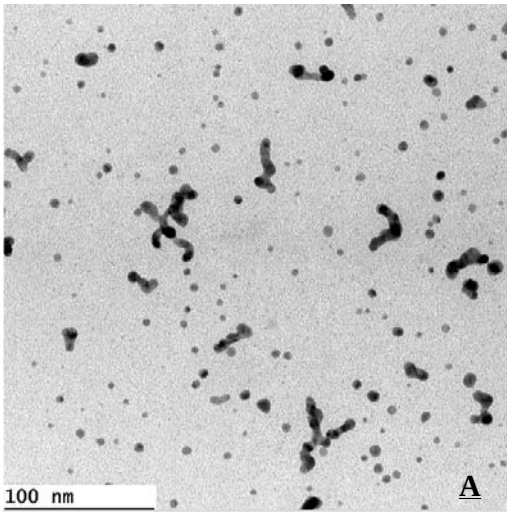

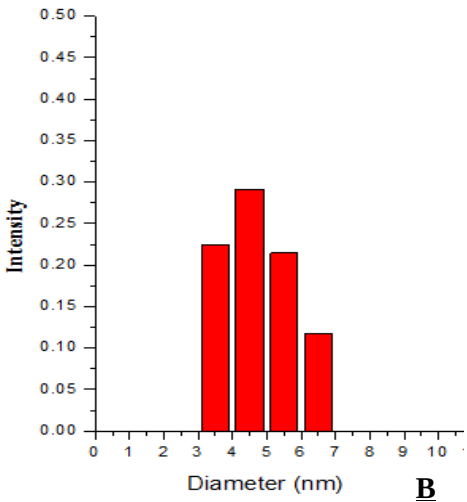

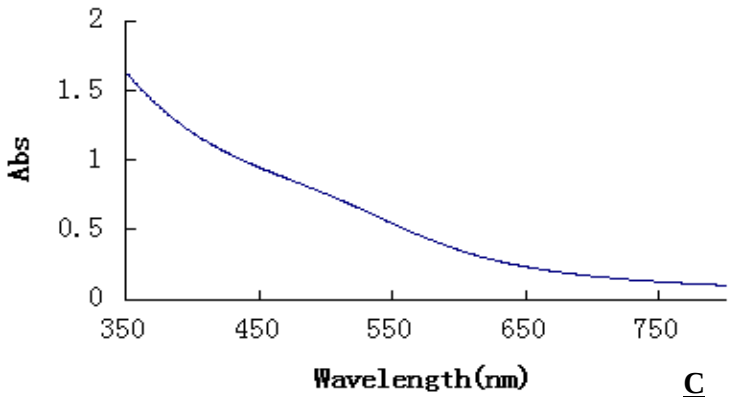

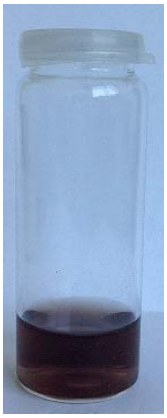

Supplementary Figure 5. A: TEM image of AuNP-**1**

B: Diameter histogram distribution of AuNP-**1** (4.5 ± 0.1 nm).

C : UV-vis. spectrum of AuNP-**1** (SPB at 512 nm)

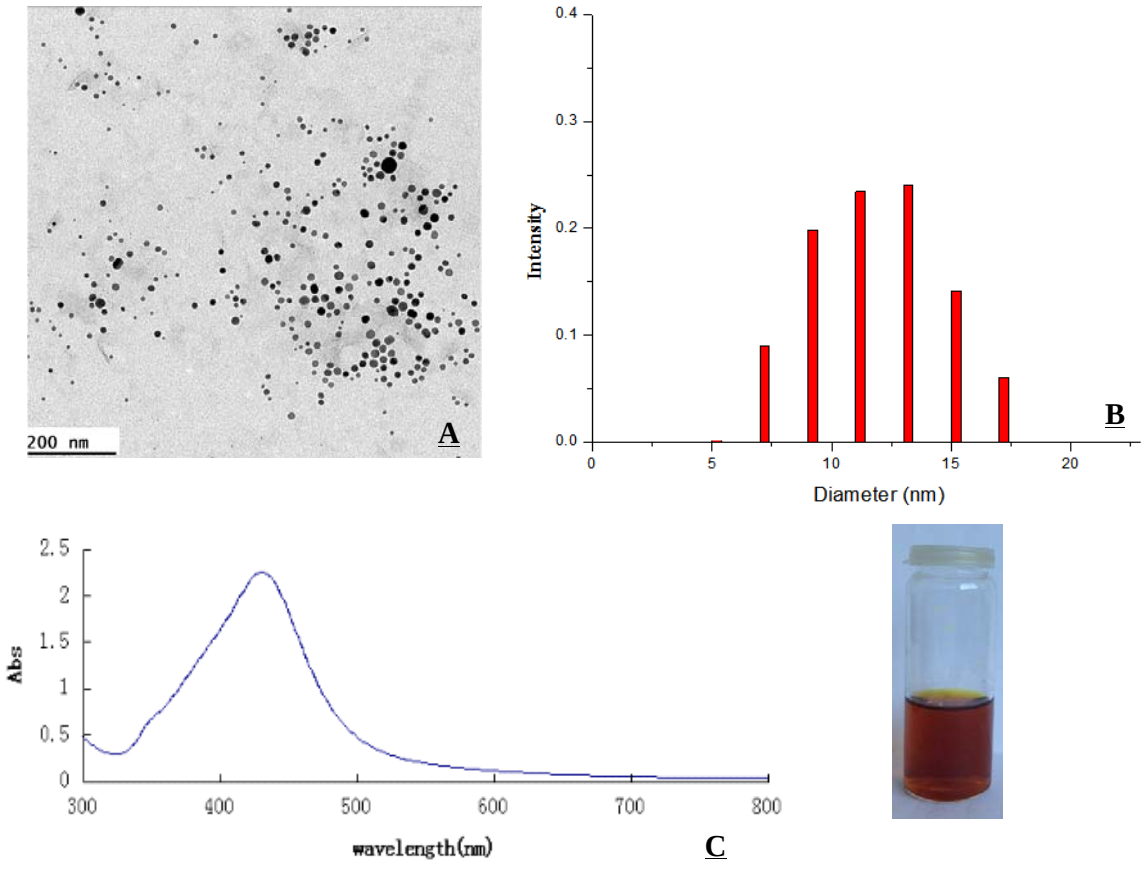

Supplementary Figure 6. A: TEM image of AgNP-**2**

B: Diameter histogram distribution of AgNP-**2** (10.9 ± 0.2 nm). .

C : UV-vis. spectrum of AgNP-**2** (SPB at 425 nm)

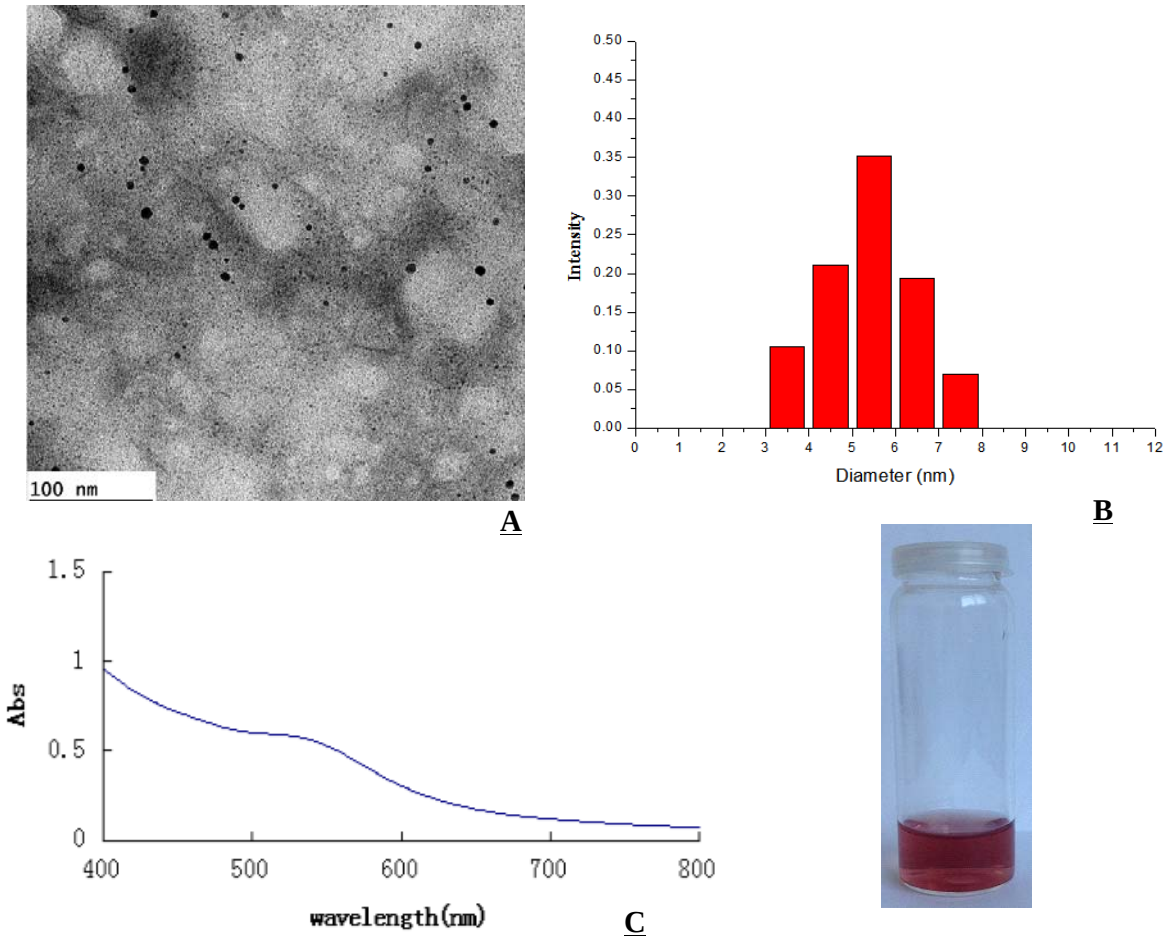

Supplementary Figure 7. A: TEM image of AuNP-**2**

B: Diameter histogram distribution of AuNP-**2** (5.1 ± 0.1 nm).

C : UV-vis. spectrum of AuNP-**2** (SPB at 540 nm)

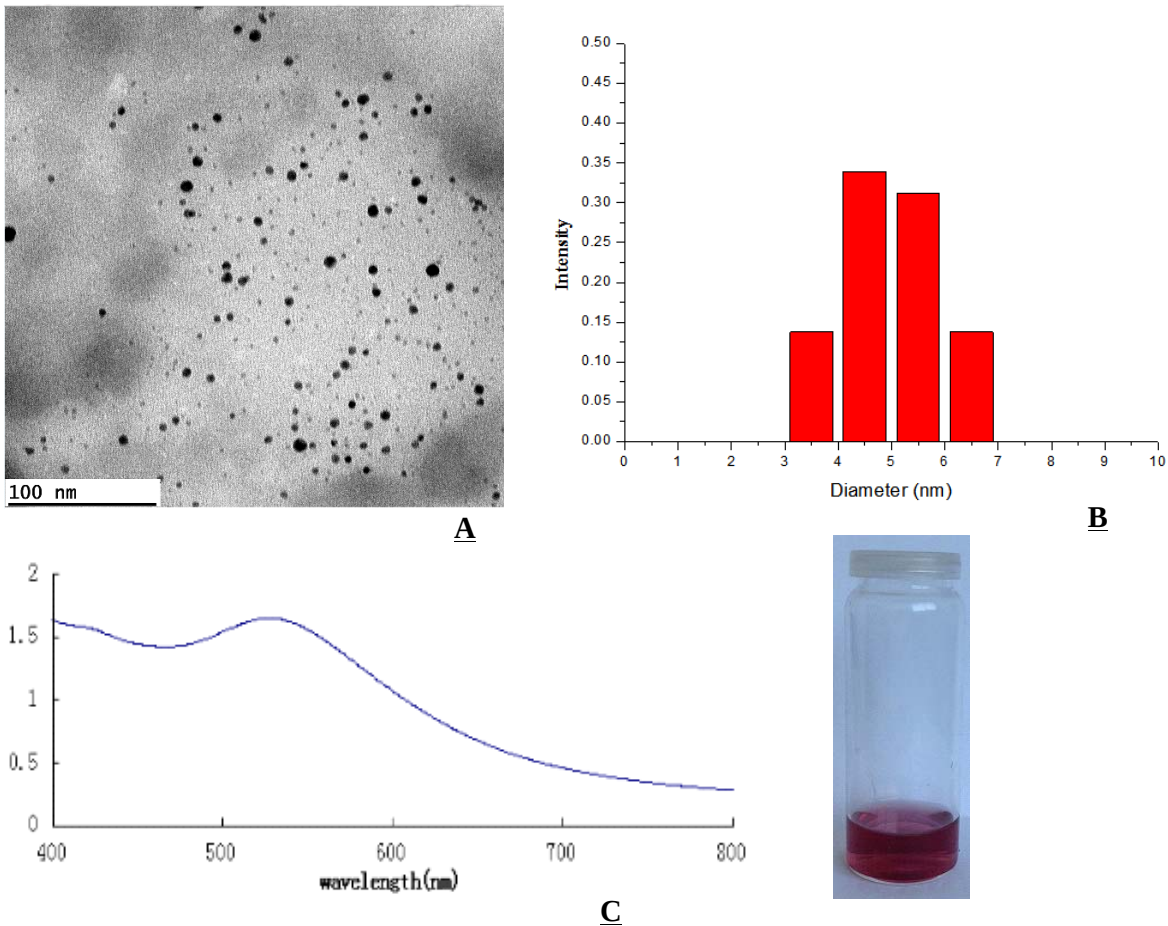

Supplementary Figures 8. A: TEM image of AuNP-**3**

B: Diameter histogram distribution of AuNP-**3 (** 4.5 ± 0.1 nm).

C : UV-vis. spectrum of AuNP-**3** (SPB at 527 nm)

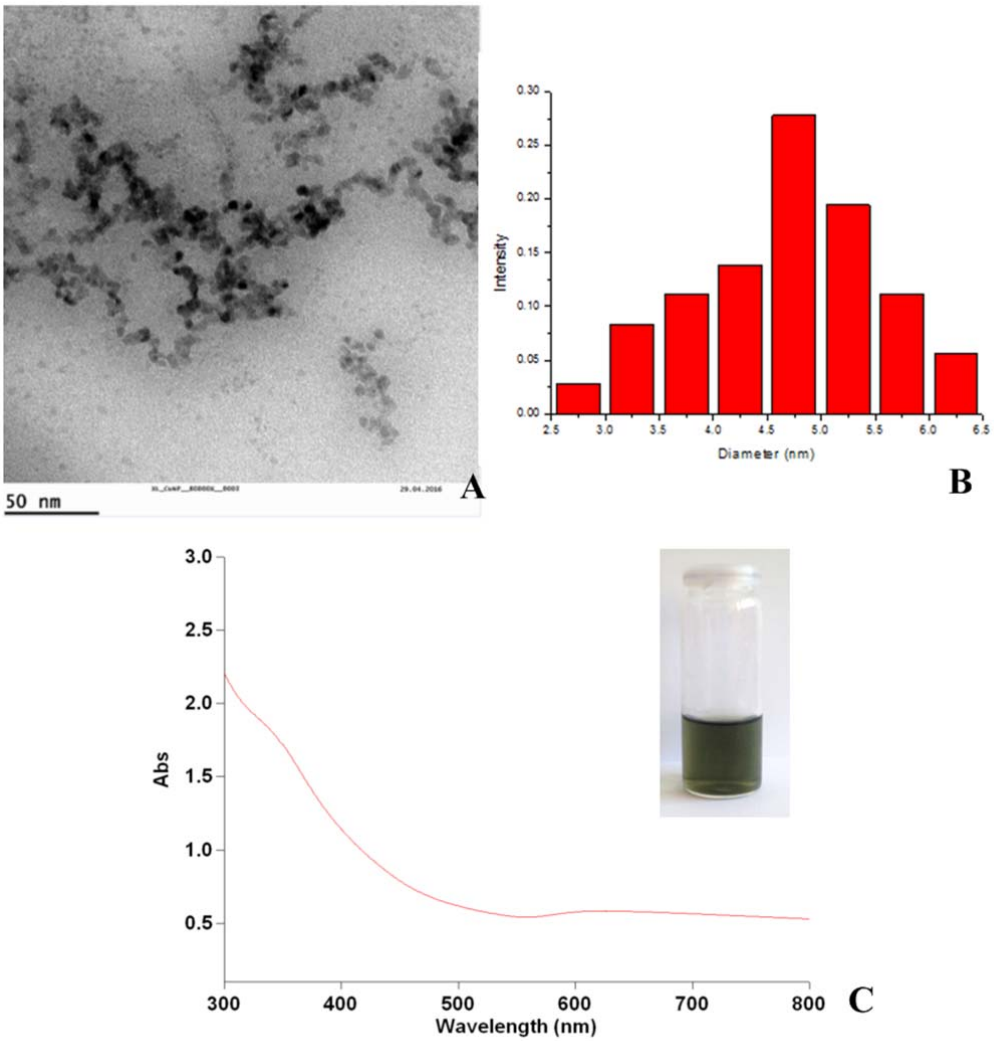

Supplementary Figure 9. A: TEM image of CuNP-**2**,

B: Diameter histogram distribution of CuNP-**2** (4.3 ± 0.1 nm).

C : UV-vis. spectrum of CuNP-**2.** (SPB at 352 and 626 nm)

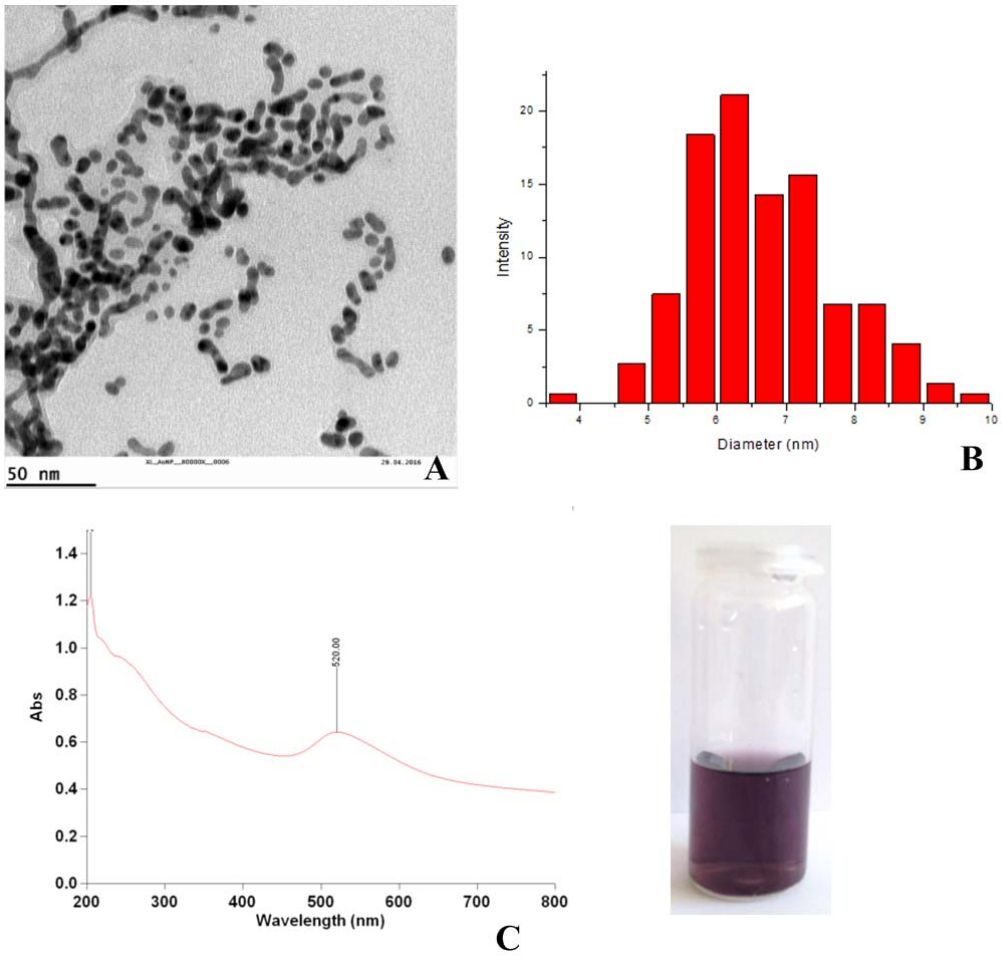

Supplementary Figure 10. A: TEM image of AuNP-**4.**

B: Diameter histogram distribution of AuNP-**4 (**6.6 ± 0.2 nm).

C : UV-vis. spectrum of AuNP-**4** (SPB at 520 nm)

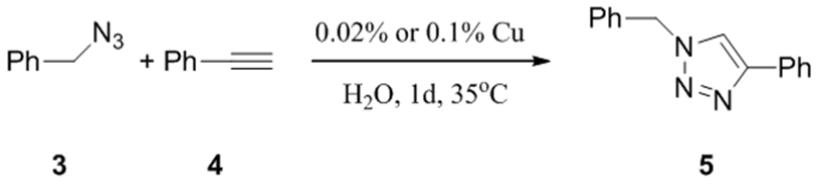

3 **4** **5**

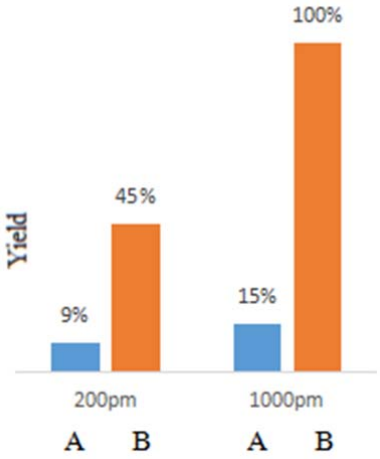

Supplementary Figure 11: Catalytic CuNP activity in the click reaction. A: CuNP-**2**. B: CuNP-**1**.

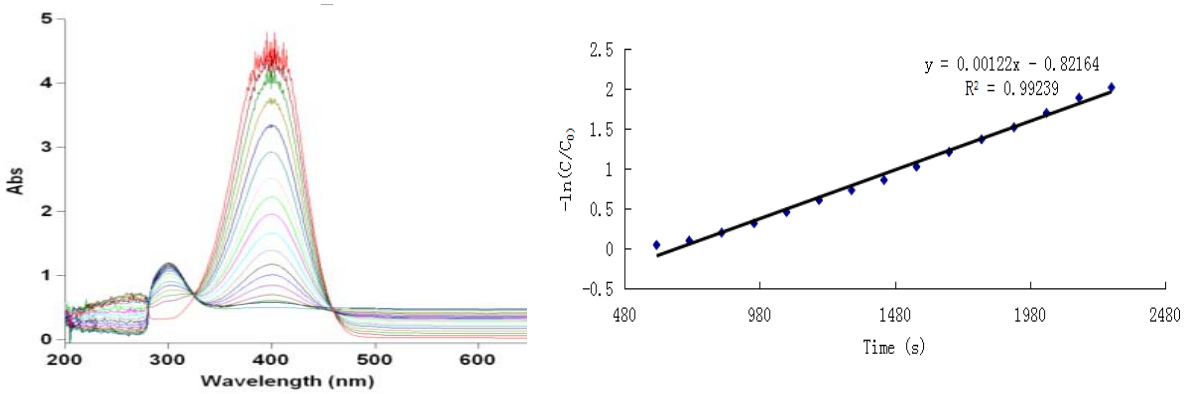

Supplementary Figure 12. 0.5 mol% AuNP-1 as catalyst for the reduction of 4-nitrophenol. a) UV-vis. spectra for the reaction monitored every min (at 22°C). **b**) Consumption rate of 4-nitrophenol: $-\ln(C/C_0)$ versus reaction time.

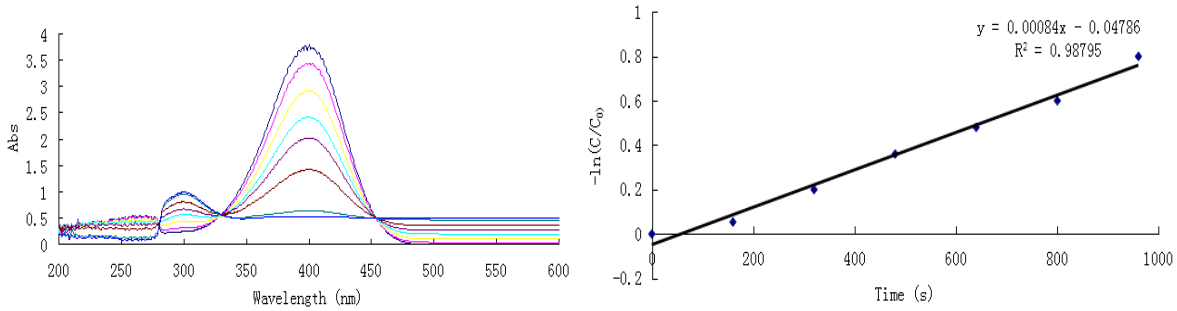

Supplementary Figure 13. 0.5 mol% AuNP-2 as catalyst for the reduction of 4-nitrophenol. a) UV-vis. spectra for the reaction monitored every min (at 22°C). **b**) Consumption rate of 4-nitrophenol: $-\ln(C/C_0)$ versus reaction time.

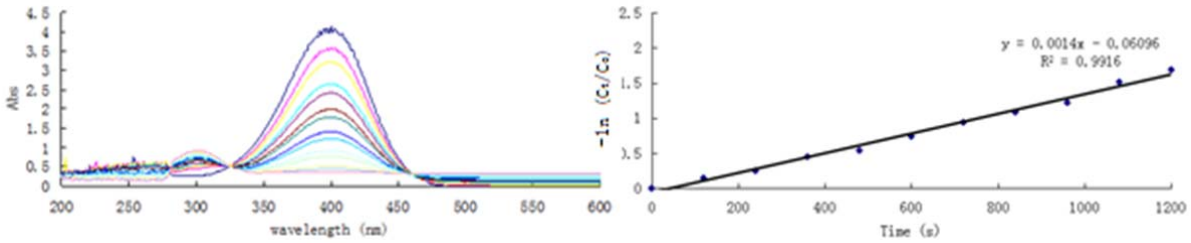

Supplementary Figure 14. 0.5 mol% AuNP-3 as catalyst for the reduction of

4-nitrophenol. a) UV-vis. spectra for the reaction monitored every min (at 22°C). **b**) Consumption rate of 4-nitrophenol: $-\ln(C/C_0)$ versus reaction time.

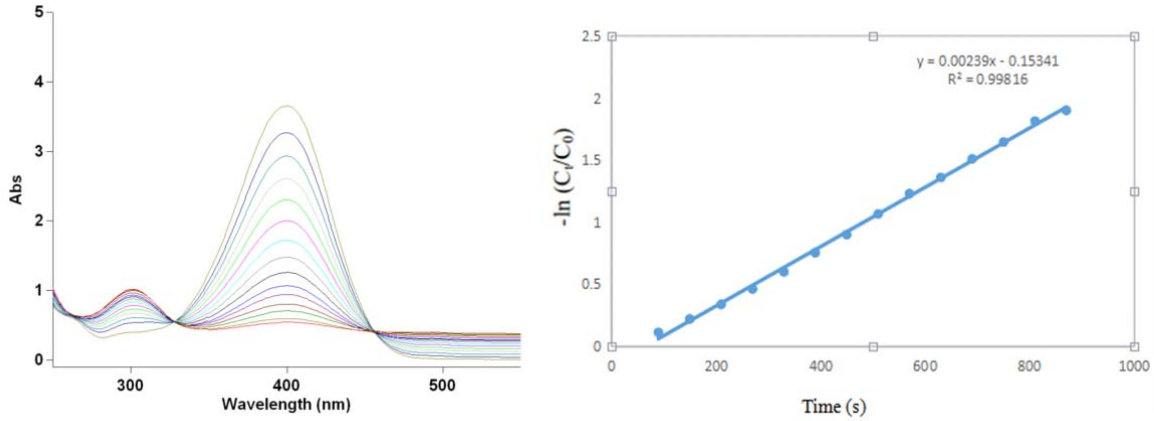

Supplementary Figure 15. 0.5 mol% AuNP-4 as catalyst for the reduction of 4-nitrophenol. a) UV-vis. spectra for the reaction monitored every min (at 22°C). **b**) Consumption rate of 4-nitrophenol: $-\ln(C/C_0)$ versus reaction time.

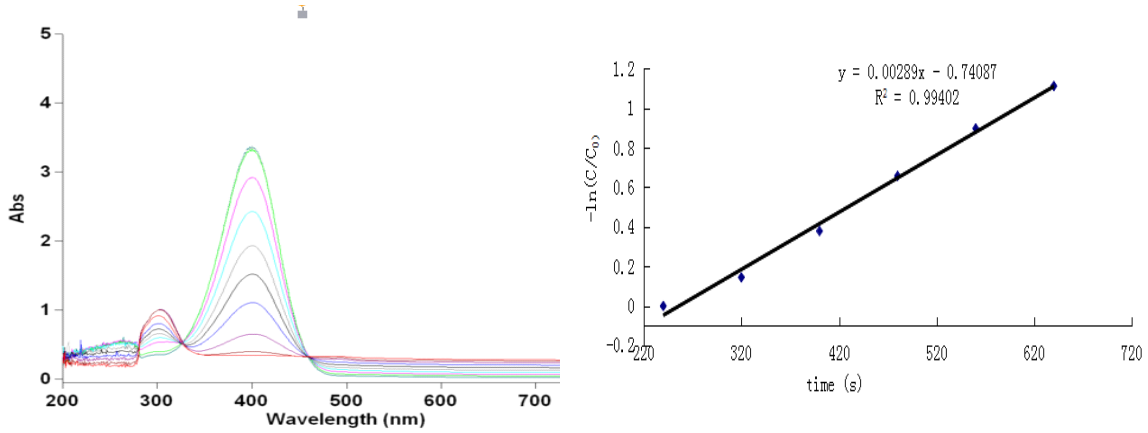

Supplementary Figure 16. **2.0 mol% AuNP-1 as catalyst for the reduction of 4-nitrophenol. a**) UV-vis. spectra for the reaction monitored every min (at 22°C). **b**) Consumption rate of 4-nitrophenol: $-\ln(C/C_0)$ versus reaction time.

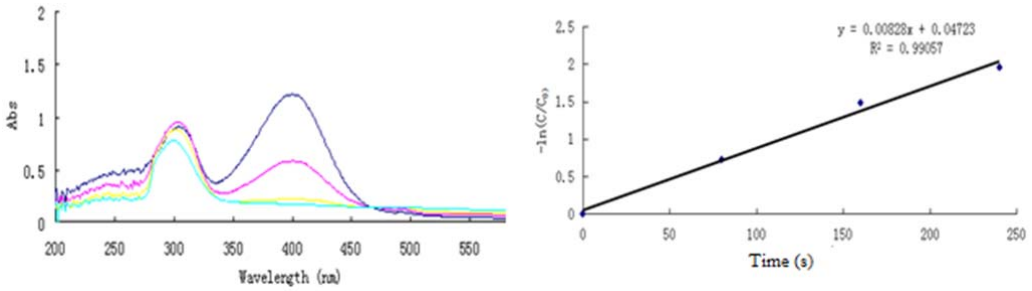

Supplementary Figure 17. 2.0 mol% AuNP-2 as catalyst for the reduction of 4-nitrophenol. a) UV-vis. spectra for the reaction monitored every min (at 22°C). **b)** Consumption rate of 4-nitrophenol: $-\ln(C/C_0)$ versus reaction time.

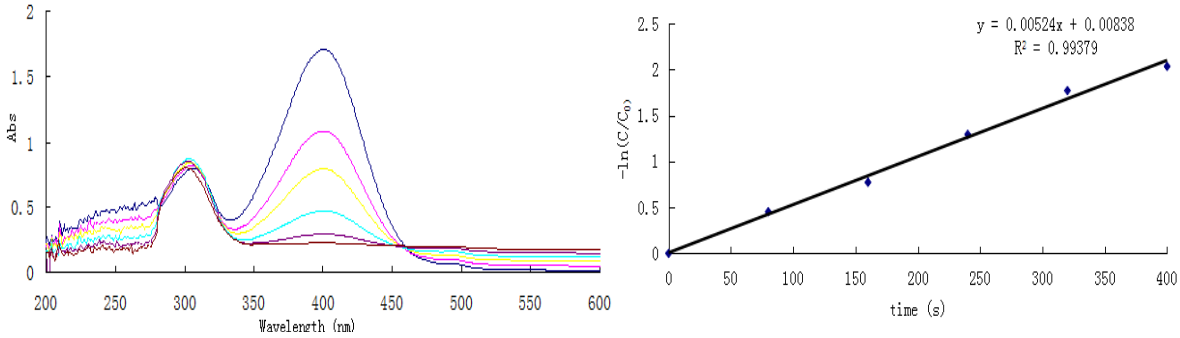

Supplementary Figure 18. 2.0 mol% AuNP-3 as catalyst for the reduction of 4-nitrophenol. a) UV-vis. spectra for the reaction monitored every min (at 22°C). **b**) Consumption rate of 4-nitrophenol: $-\ln(C/C_0)$ versus reaction time.

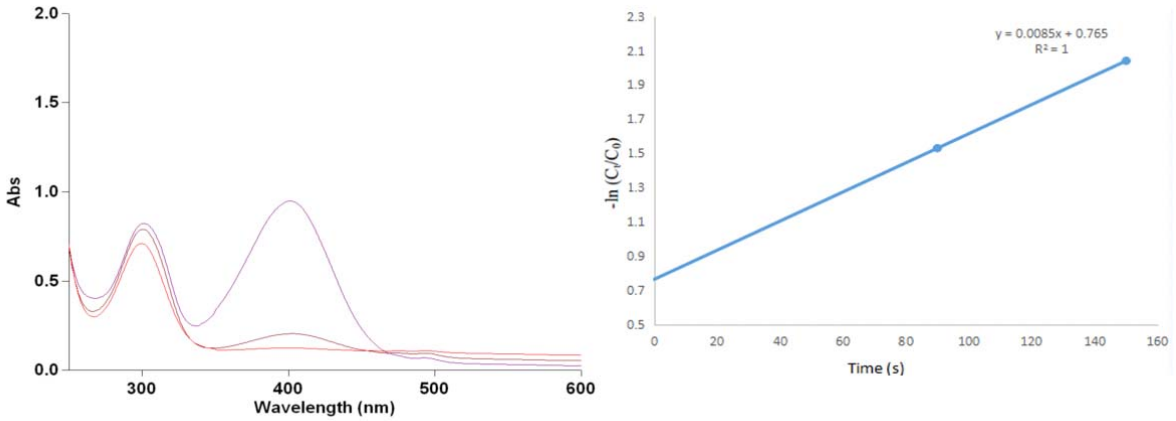

Supplementary Figure 19. 2.0 mol% AuNP-4 as catalyst for the reduction of 4-nitrophenol. a) UV-vis. spectra for the reaction monitored every min (at 22°C). **b**) Consumption rate of 4-nitrophenol: $-\ln(C/C_0)$ versus reaction time.

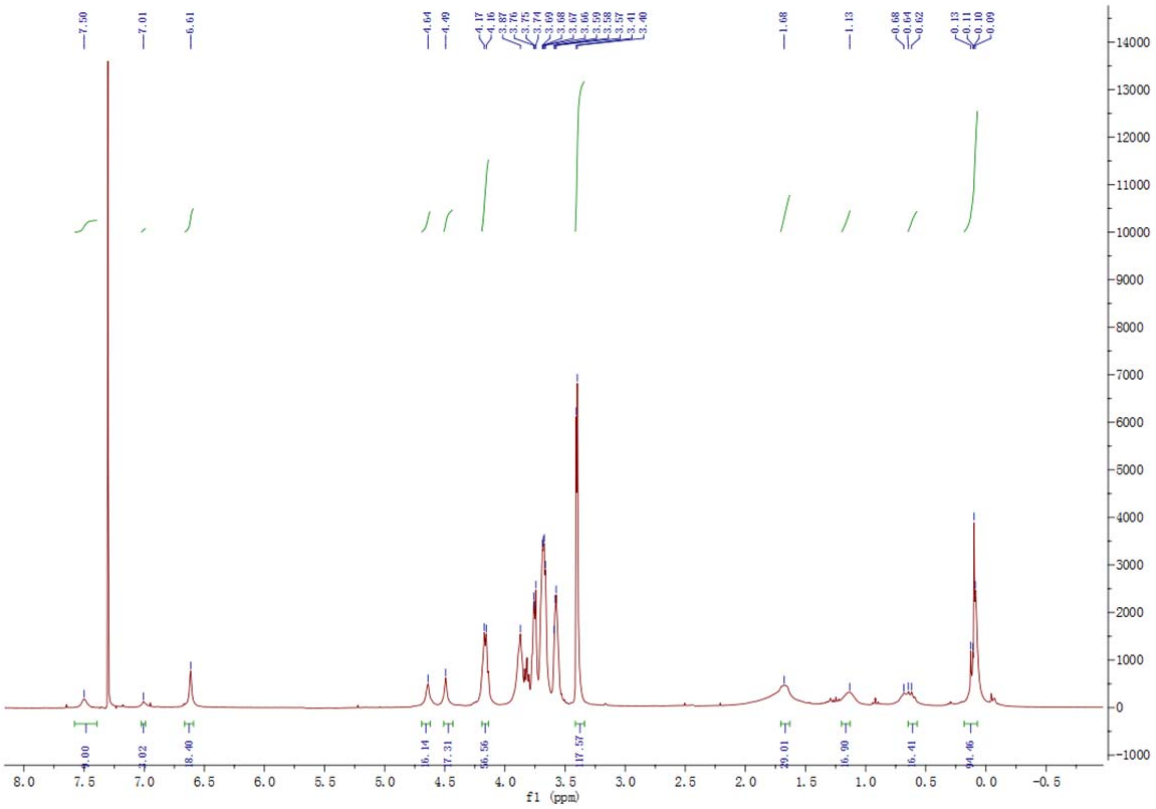

Supplementary Figure 20: ^{1}H NMR spectrum of dendrimer **1** in $CDCl_3$.

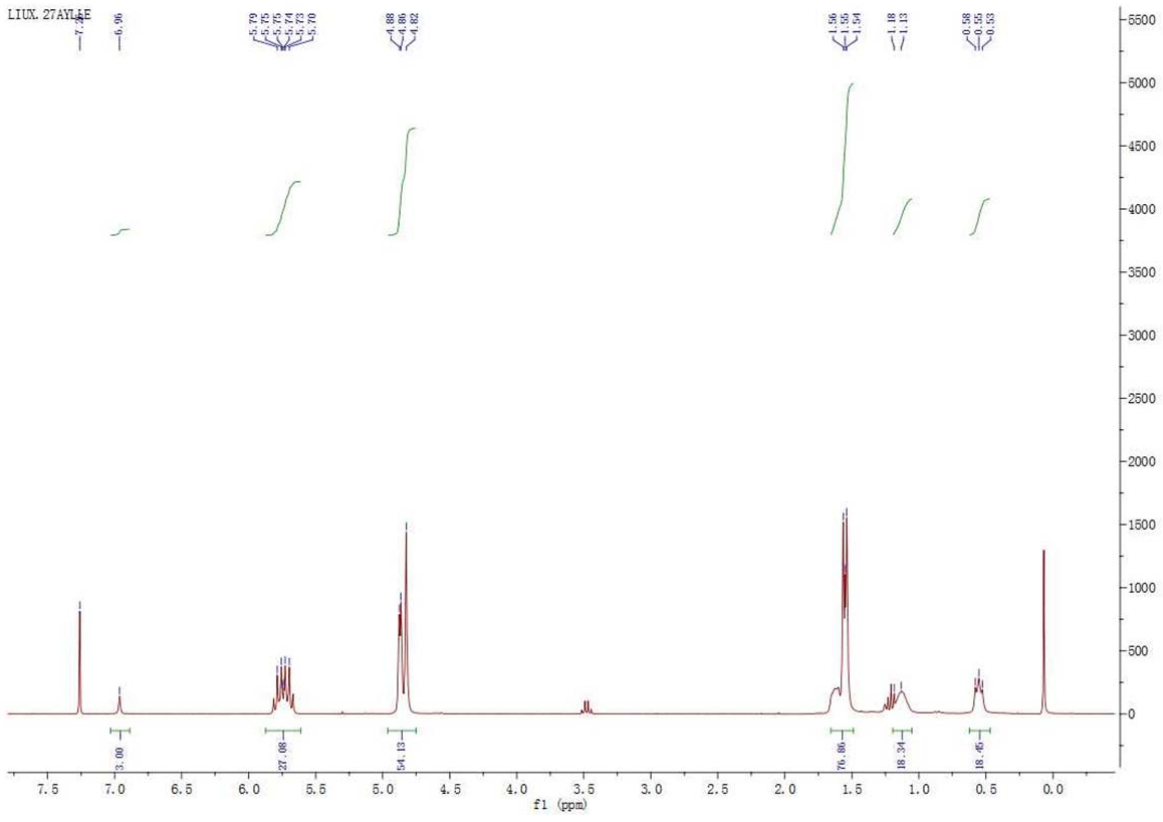

Supplementary Figure 21: ^{1}H NMR spectrum of dendrimer **2** in $CDCl_3$.

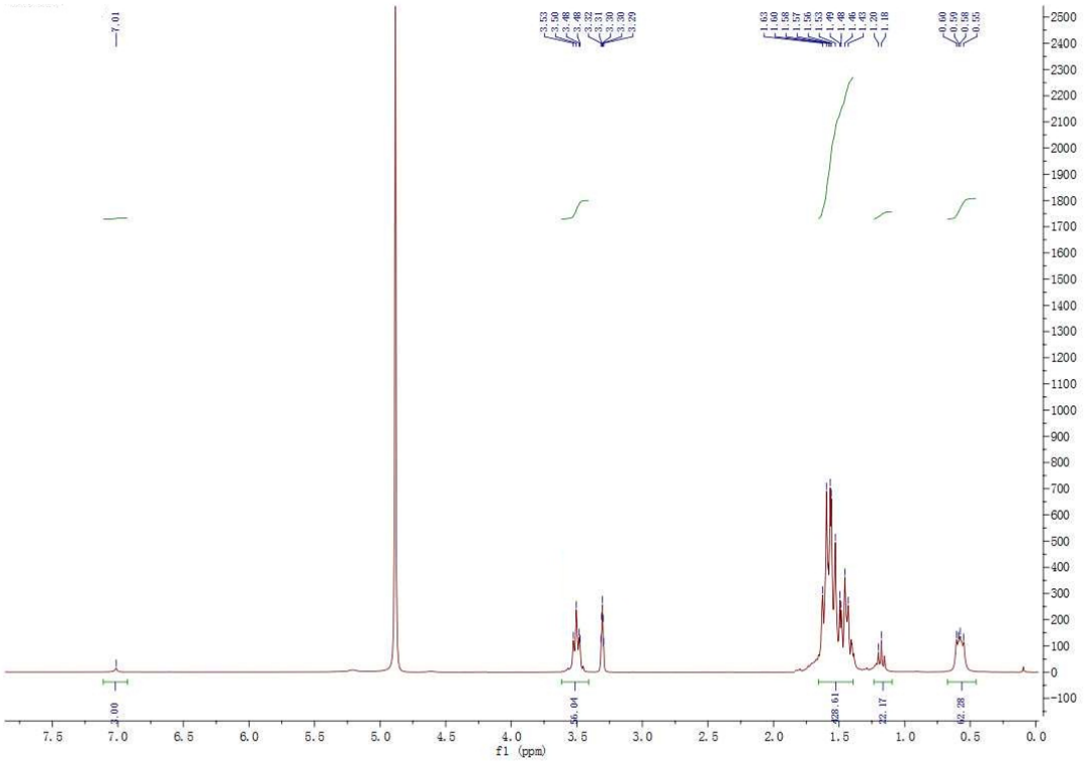

Supplementary Figure 22: ^{1}H NMR spectrum of dendrimer **2** in MeOD.

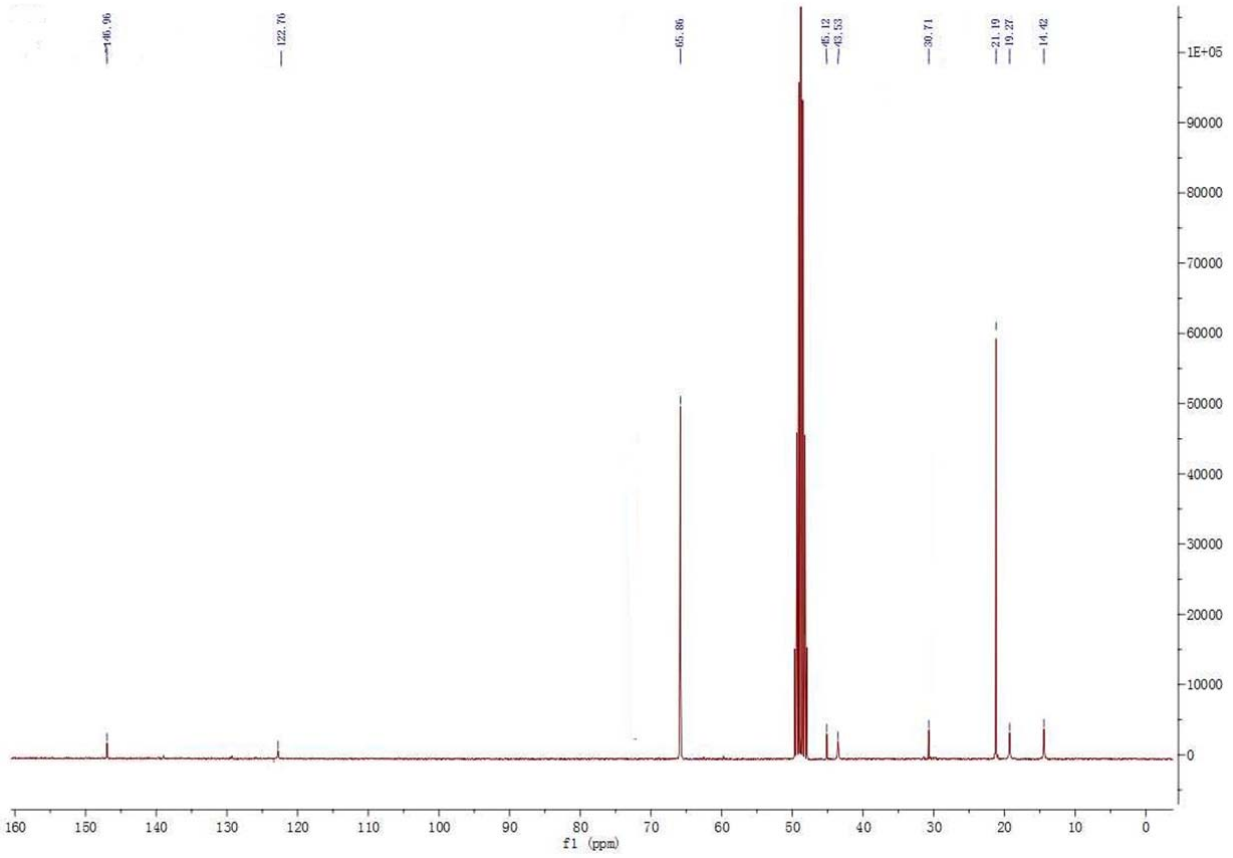

247

248 **Supplementary Figure 23**: ^{13}C NMR spectrum of dendrimer **2** in MeOD

249

250

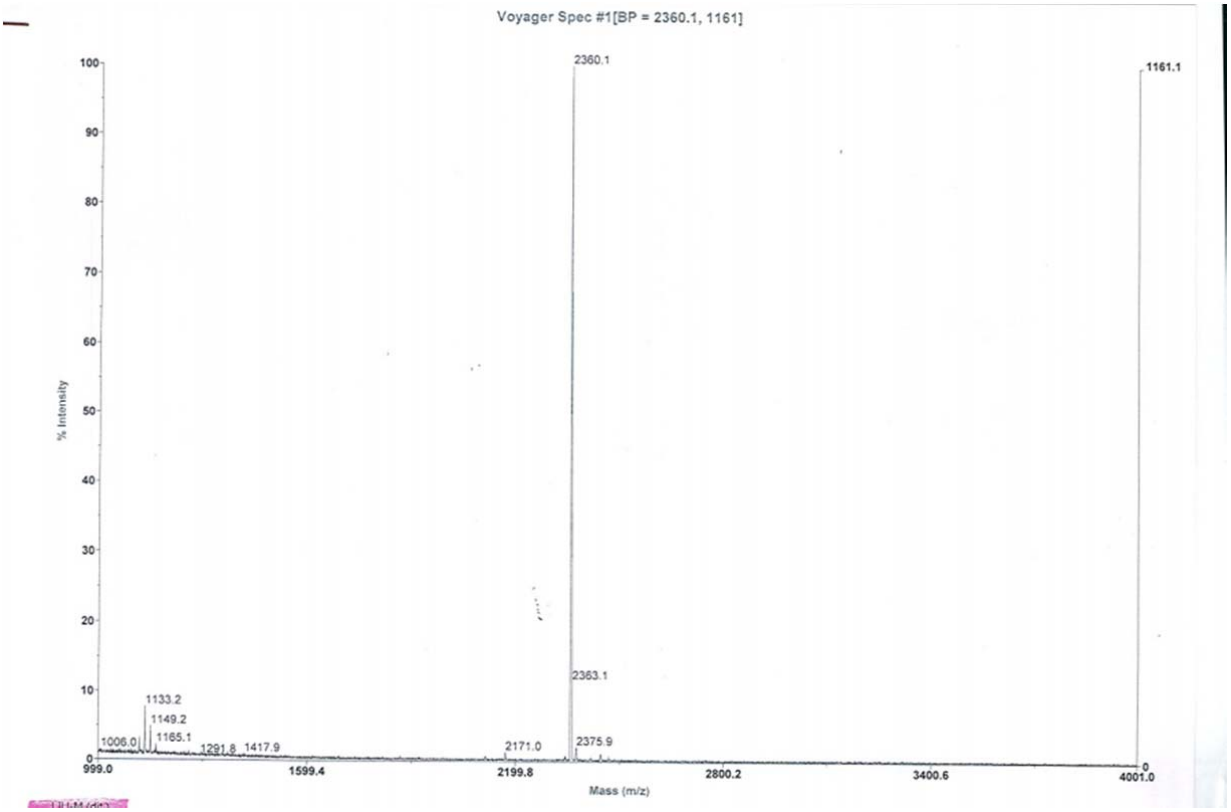

Supplementary Figure 24: Mass spectrum of dendrimer **2**. ESI HRMS: calcd. for $C_{117}H_{246}O_{27}Si_9$ $[M+Na]^+$: 2360.1386, found: 2360.1116.

The mass spectra were recorded at the CESAMO (Univ. Bordeaux) with a QStar Elite

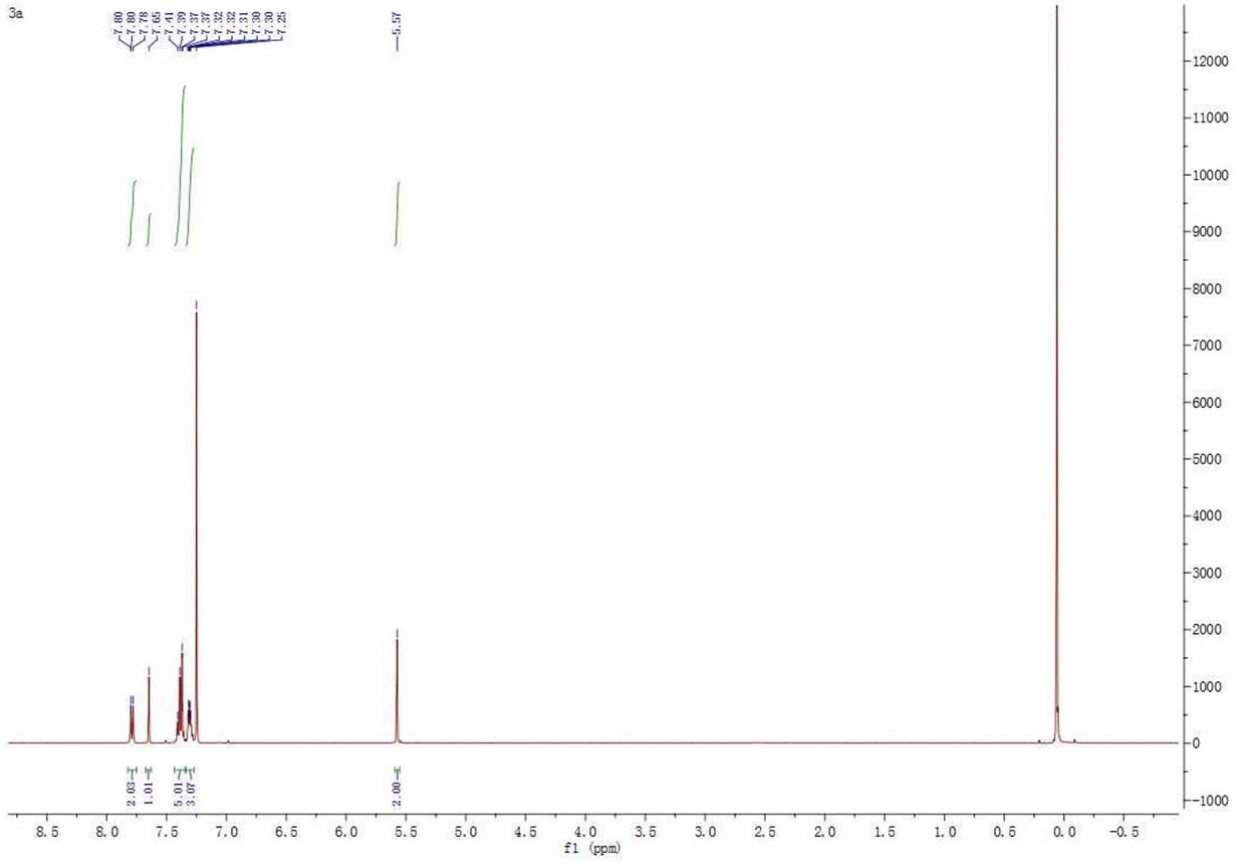

Supplementary Figure 25: ^{1}H NMR spectrum of **5** in $CDCl_3$.

Supplementary Table 1. Catalytic AuNPs activity in the reduction of 4-NP [a]

	T_0 (s)	T (s)	k_a (s^{-1})	D (nm)
2.0 mol % AuNP-**1**	180	880	$2.8*10^{-3}$	4.5
2.0 mol % AuNP-**2**	0	240	$8.1*10^{-3}$	5.1
2.0 mol % AuNP-**3**	0	400	$5.1*10^{-3}$	4.5
2.0 mol% AuNP-**4**	0	210	$8.5*10^{-3}$	6.6
0.5 mol % AuNP-**1**	480	1860	$1.1*10^{-3}$	4.5
0.5 mol % AuNP-**2**	0	1260	$1.6*10^{-3}$	5.1
0.5 mol % AuNP-**3**	0	1560	$1.4*10^{-3}$	4.5
0.5 mol % AuNP-**4**	0	1200	$2.4*10^{-3}$	6.6

[a] T_0 : induction time. T : reaction time. D: diameter of nanoparticles

AuNP-**1** Reduction of the Au^{III} to AuNP by $NaBH_4$ Dendrimer **1**

AuNP-**2** Reduction of the Au^{III} to AuNP by CuNP Dendrimer **1**

...

Supplementary Discussion

Copper chemical state differentiation can be difficult with XPS only due to overlap of binding energies, especially when distinguishing Cu (I) and Cu (0). In the 2p region Cu 2p3/2 peak in Cu (I) oxide is not shifted but broader compared to Cu metal. We have found our main 2p 3/2 peak assigned to one of these states at binding energy of 933.45 eV as shown in Figure 6B. It is established that Cu2p3/2 peak in Cu (II) oxide is shifted, and in our spectra (Figure 6B) we found this peak shifted for 1.45 eV from Cu(0)/Cu(I) position. This is an observable value considering our spectral resolution of 1.1 eV and following the fitting of our peaks with reported Gaussian-Lorentzian 80-20 and Gaussian- Lorentzian 90-10 peak shapes, resulting fit clearly represents a mixture of Cu (0)/Cu(I) and Cu (II) species. [3]

Several authors have reported how it is feasible to resolve Cu oxidation states using satellite features of Cu 2p, especially when it is needed to distinguish Cu (II) from Cu (0) /Cu(I) as in our case. [2-8].

Cu (II) has an observable collection of satellite features at 943 eV, and we have found these at BE of 941.29 and 944.93 eV, which is in nice agreement with work collected and reported in the review of Biesinger et al.[9] In this paper a quantification procedure for Cu species incorporating the use of the shake-up satellites for Cu(II)-containing compounds is elaborated based on a numerous literature and reports of peak FWHM, BE and peak shape. Furthermore the same analysis approach is applied in additional literature describing how a strong shake-up satellite is observed for CuO, as shown in Figure 6B but absent for Cu_2O and metallic copper.[10,11] A detailed examination of line shape and shake up satellite feature found at 963.25 eV strengthens our claim as this satellite is reported only for the Cu (II) state. (9,10)

Still there is the possibility of coexistence of Cu(0) and Cu(I) or Cu(I) alone. While this may be resolved by other complementary techniques, our evidence is strong in oxidation of Cu to Cu (II).

On the other hand, for CuNP-1, pure Cu (0) shown in Figure 6A does not show shake

up at 943 eV, and it is not present at 963.5 eV, which clearly confirms that it is pure Cu(0). If Cu (I) would be present, a weak satellite feature would be recorded at these energies, which is not case here. [2-11]

Supplementary Methods

General Analytical Information.

All commercial materials were used without further purification, unless indicated. ^{1}H NMR spectra were recorded on a Bruker Avance 300 FT (^{1}H: 300MHz, ^{13}C: 75.3MHz) spectrometer. The chemical shifts for the NMR spectra are reported in ppm relative to the solvent residual peak.[1] Coupling constants J are reported in hertz (Hz). The following abbreviations are used for the multiplicities: s, singlet; d, doublet; t, triplet; q, quartet; qt, quintet; st, sextet; m, multiplet; br, broad; dd, doublet of doublet. Yields refer to isolated material determined to be pure by NMR spectroscopy and thin-layer chromatography (TLC), unless specified in the text. Flash chromatography was performed on Fluka Silica Gel 60 Å, 40-63 μm. Milli-Q water (18.2 MΩ) was used in the preparation of AuNPs. XRD measurements were performed by Burker D8 Advance Diffractometer (monochromatic Cu radiation, LynxEye detector). The mass

spectra were recorded at the CESAMO (Univ. Bordeaux) with a QStar Elite mass spectrometer (Applied Biosystems). UV-vis. absorption spectra were measured with a Perkin-Elmer Lambda 19 UV-vis. spectrometer. Elemental analyses were recorded on a PAR 273 potentiostat under nitrogen atmosphere. The DLS measurements were made using a Malvern Zetasizer 3000 HSA instrument at 25° C at an angle of 90°. Transmission Electron Microscopy (TEM) images of nanoparticles were obtained on a JEOL JEM 1400 (120 kV) microscope. The TEM samples were prepared by deposition of the nanoparticle suspension (10 µ L) onto a carbon-coated microscopy copper grid. Samples were left to dry in vacuum prior to insertion into TEM microscope. Scanning-Transmission Electron microscopy (STEM) images were recorded by FEI TITAN Low-base equipped with a CESCOR Cs-probe corrector (CEOS, Heidelberg, Germany) in high angle annular dark field. EDX spectra are recorded with an energy resolution of 0.14 eV using Gatan Energy Filter Tridiem 866 ERS and a monochromator at 300 kV. X-ray Photoelectron Spectroscopy (XPS) Surface analysis by XPS was performed in a SPECS SAGE HR 100 system spectrometer in an ultra-high vacuum (UHV) chamber. The X-ray sources employed for this analysis were an non monochromatic Mg K^{α} (1253.6 eV) and 250 W or Al K^{α} operated at 1.25 kV and 300W, calibrated using the $3d_{5/2}$ line of Ag with a full width at half maximum (FWHM) of 1.1 eV. The take- off angle was fixed at 90° and the analysis was conducted at a pressure of $\sim 10^{-6}$ Pa. Samples were dispersed onto glass, silicon or titanium surfaces, dry and inserted into the XPS The selected resolution for the spectra was 30 eV of Pass Energy and 0.5 eV/step for the general survey spectra and 15 eV of Pass Energy and 0.15 eV/step for the detailed spectra of Cu 2p, Ag 3d and Au 4f photoelectron lines. Spectra were analyzed with the CasaXPS 2.3.15dev87 software. The analysis consisted of satellite removal, Shirley background subtraction, calibration of the binding energies related to the C 1s C-C peak at 285 eV, and peak fitting with Gaussian-Lorentizan line shapes where the FWHM of the peaks were constrained while the peak positions and areas were set free.

Preparation of dendrimer 1

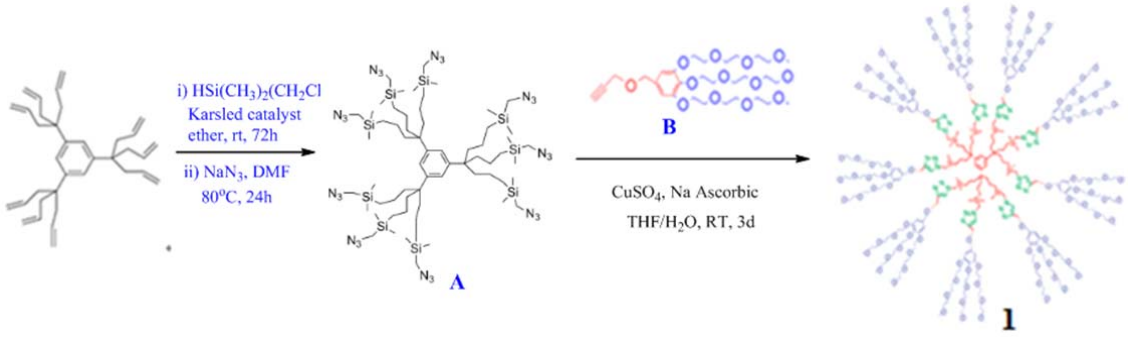

Dendrimer **1** has been synthesized following reference 31 of the main text. 9-azido core (**A**) (0.012 mmol) and the alkyne tris TEG dendron (**B**)(0.13 mmol, 1.2 equiv. per branch) are dissolved in THF. $CuSO_4.5H_2O$ is added (0.032 g, 0.13 mmol, 1 equiv. per branch, 1M in aqueous solution), followed by dropwise addition of a freshly prepared solution of sodium ascorbate (0.051 g, 0.26 mmol, 2 equiv. per branch, 1M in water solution) in order to set a 1:1 THF/water ratio. The reaction mixture is stirred for 3 days at 25°C under N_2. After removing THF *in vacuo*, CH_2Cl_2 (100 mL) and an aqueous ammonia solution (2.0 M, 50 mL) are successively added. The mixture is allowed to stir for 10 minutes in order to remove all the Cu^{II} trapped inside the dendrimer as $[Cu(NH_3)_2(H_2O)_2]$ $[SO_4]$. The organic phase is washed twice with water, then this operation is repeated three more times to ensure complete removal of copper ions. The organic phase is dried with sodium sulfate, and the solvent is removed *in vacuo*. The product is washed with 50 mL diethyl ether several times in order to remove the excess of the dendron. Dendrimer **1** is obtained (in 68 % yield).

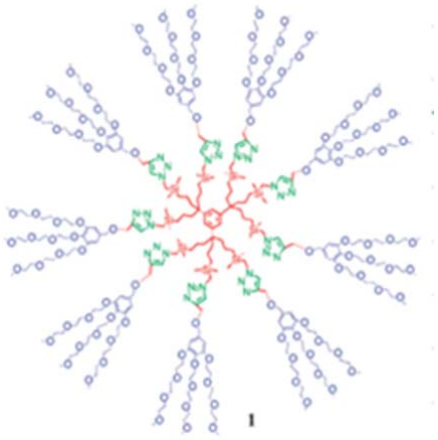

1

^{1}H NMR ($CDCl_3$, 300 MHz) δ: ppm: 7.50 (CH-triazole), 7.00 (CH-arom. intern), 6.61 (CH- arom. extern), 4.54 (triazole-CH_2-0), 4.40 (O-CH_2-arom. extern), 4.04-4.09 (CH_2O-arom. extern), 3.90 (Si-CH_2-triazole), 3.51-3.78 (OCH_2CH_2O), 3.49-3.42 (CH_3O), 1.68 ($CH_2CH_2CH_2Si$), 1.13 ($CH_2CH_2CH_2Si$), 0.62 ($CH_2CH_2CH_2Si$), 0.09 ($Si(CH_3)_2$). As show in Supplementary Fig.20.

Preparation of dendrimer 2

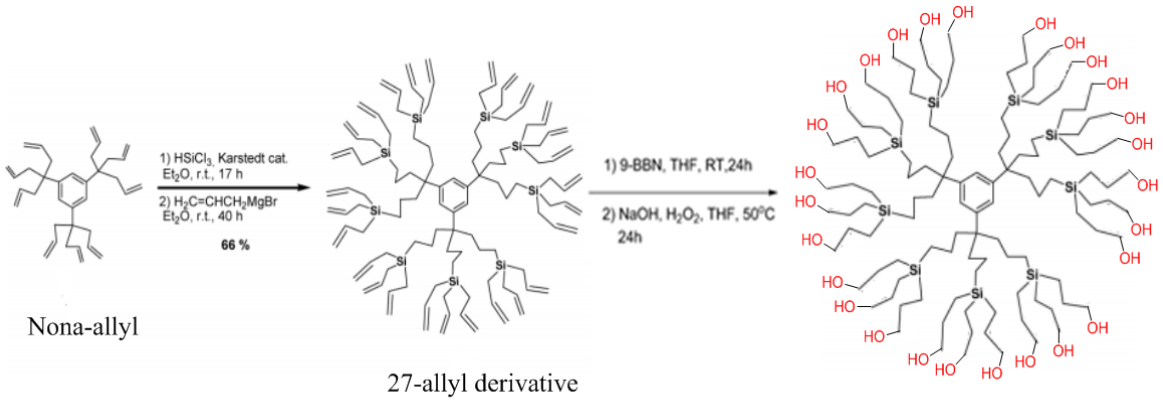

Procedure for the preparation of the 27-allyl dendrimer

The nona-allyl (0.20 g, 0.42 mmol) and trichlorosilane (1.5 mL, 11.34 mmol, 3 equiv per branch) were dissolved in Et_2O (20 mL), and 10 drops of Karstedt catalyst were added. After being stirred for 17 h at room temperature (rt), the mixture was

concentrated to dryness and the residue redissolved in Et_2O (20 mL). Then, a solution of allylmagnesium bromide in Et_2O (1M, 34 mL, 34.02 mmol, 9 equiv per branch) was added dropwise at 0°C. After the micture was stirred for 48 h at rt, an aqueous solution of NH_4Cl (20 mL) and water (20 mL) were added dropwise, successively, at 0 °C. The organic phase was washed with water (3 × 100 mL), dried over Na_2SO_4, filtered, and concentrated to dryness. The crude product was dissolved in a minimum amount of Et_2O and precipitated with an excess of methanol. The 27-allyl derivative was obtained as a colorless oil in 66% yield (0.51 g).

Procedure for the preparation of dendrimer **2**.

The 27-allyl (0.27mmol) dissolved in 40 mL of freshly distilled THF, then is introduced into a flamed Schlenk tube under nitrogen, then 9-BBN (0.5 M in THF, 80 mL) was added at 0^oC. And the reaction mixture is stirred for one day at room temperature in the dark. Then, 30 mL degassed water, 30 mL 3M NaOH and 45 mL 30% H_2O_2 are added. The reaction mixture is left 15h at 50^oC, the two phases are separated, the aqueous phase is saturated with potassium carbonate and extracted with 5 x 50 mL THF. The organic phase are gathered and dried over sodium sulfate, filtered, and concentrated to 30 mL under reduced pressure. The dendrimer **2** is obtained by precipitation upon addition of 100 mL ether. (0.512g, 84% yield).

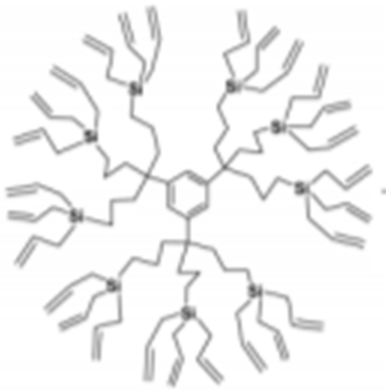

27-allyl-terminated dendrimer ^1H NMR ($CDCl_3$, 300 MHz) δ: ppm: 6.98 (br, 3H, CH of arom. core), 5.76 (m, 27H, $SiCH_2CH=CH_2$), 4.87 (m, 54H, SiCH2CH=CH2), 1.57 (br, 72H,$CH_2CH_2CH_2Si$, $SiCH_2CH=CH_2$), 1.18 (br, 18H, $CH_2CH_2CH_2Si$), 0.55 (br, 18H, $CH_2CH_2CH_2Si$). As show in Supplementary Fig. 21.

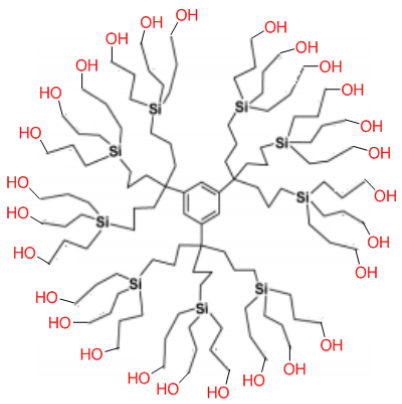

2

^{1}H NMR (MeOD, 300 MHz) δ: ppm: 7.04 (s, 3H) (CH-arom.intern), 3.50 (m, 54H) (CH_2OH extern), 1.14-1.16 (CH_2.CH_2), 0.60 CH_2Si). As show in Supplementary Fig. 22, ^{13}C NMR (MeOD, 75MHz) δ: ppm: 148.9, 122.7, 66.8, 45.1, 43.5, 30.7 21.2, 19.3, 14.42. As show in Supplementary Fig. 23. ESI HRMS: calcd. for $C_{117}H_{246}O_{27}Si_9$ $[M+Na]^+$: 2360.1386, found: 2360.1116. As show in Supplementary Figure 24. anal. calcd. for $C_{117}H_{246}O_{27}Si_9 + 7H_2O$: C 57.03, H 10.50 ; found: C 57.05, H 9.99.

Procedure for the preparation of 5

Azidomethylbenzenel (**3**) 0.5 mmol, ethynylbenzene (**4**) 0.55 mmol, CuNP-**2** (0.1% mmol) and 2ml H_2O were taken in a round bottom flask equipped with stirrer. The resulting mixture was stirred for 24 h at 35°C under N_2. After cooling to rt, water (2 mL) was added to the reaction mixture, and the latter was extracted with ethyl acetate (3×10 mL). The combined organic phases were washed with brine (2×5 mL), dried over anhydrous $MgSO_4$ and concentrated *in vacuo*. The residue was subjected to flash column chromatography with hexanes/EtOAc (v/v = 5/1) as eluent to obtain the desired 1-benzyl-4-phenyl-1H-1,2,3-triazole (**5)** as white solid (100% yield).

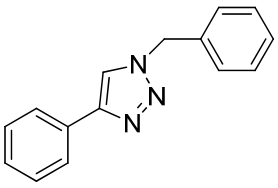

5

1-benzyl-4-phenyl-1H-1,2,3-triazole **(5)** was purified by flash chromatography (hexane-EtOAc, v/v=5/1) as white solid (yield: 98 %). ^{1}H NMR (300 MHz, $CDCl_3$): δ: 7.78-7.80 (d, J=6Hz, 2H), 7.65 (s, 1H), 7.37-7.41 (m, 5H), 7.25-7.32 (m, 3H), 5.57 (s, 2H). As show in Supplementary Fig. 25.

Supplementary References:

[1]1.-Fulmer, G. R., Miller, A. J. M., Sherden, N. H., Gottlieb, H. E., Nudelman, A., Stoltz, B. M., Bercaw, J. E. & Goldberg, K. I. NMR Chemical Shifts of Trace Impurities: Common Laboratory Solvents, Organics, and Gases in Deuterated Solvents Relevant to the Organometallic Chemist. *Organometallics.* **29**, 2176-2179. (2010**)**

2.- Tahir, D. & Tougaard, S. Electronic and optical properties of Cu, CuO and Cu2O studied by electron spectroscopy. J. Phys.: Condens. Matter. **24**,175002 (2012).

3.- Poulston, S., Parlett, P. M., Stone, P. & Bowker, M. Surface Oxidation and Reduction of CuO and Cu_2O Studied Using XPS and XAES. *Surf. Interface Anal*. **24**, 811-820 (1996).

4.- Wang, D. N., Miller, A. C. & Notis, M. R. XPS Study of the Oxidation Behavior of the Cu3Sn Intermetallic Compound at Low Temperatures. *Surf. Interface Anal.* **24,** 127-132 (1996) .

5.- Meda, L. & Cerofolini, G. F. A decomposition procedure for the determination of copper oxidation states in Cu-zeolites by XPS. *Surf. Interface Anal*. **36**, 756-759 (2004).

6.- Feng, L., Zhang, L., Evans, D. G. & Duan, X. Structure and surface chemistry of manganese-doped copper-based mixed metal oxides derived from layered double hydroxides. *Colloids Surf. A.* **244**, 169-177 (2004).

7.- Kundakovic, L. J. & Flytzani-Stephanopoulos, M. Reduction characteristics of

480 copper oxide in cerium and zirconium oxide systems. *Appl. Catal. A.* **171**, 13-29
481 (1998).

482 8.- Salvador, P., Fierrom, J. L., G. Amador, J., Cascales, C. & Rasines, I. XPS study of
483 the dependence on stoichiometry and interaction with water of copper and oxygen
484 valence states in the $YBa_2Cu_3O_{7-x}$ compound. *J. Solid State Chem.* **81**, 240-249
485 (1989).

486 9.-Biesinger, M. C., Lau, L. W. M., Gerson, A. R. & Smart, R. S. C. Resolving surface
487 chemical states in XPS analysis of first row transition metals, oxides and hydroxides:
488 Sc, Ti, V, Cu and Zn. *Appl. Surf. Sci*. **257**, 887-898 (2010).

489 10.- Biesinger, M. C., Hart, B. R., Polack, R., Kobe, B. A. & Smart, R. S. C. Analysis
490 of mineral surface chemistry in flotation separation using imaging XPS. *Miner. Eng.*
491 **20**, 152-162 (2007).

492 11.-Watts, J. F. & Wolstenholme, J. An Introduction to Surface Analysis by XPS and
493 AES, Wiley, Rexdale, p. 71 (2003).

494

495

496

497

498
